# On the Facile Synthesis of Triazaarsoles Using Acyclic Precursors

**DOI:** 10.1002/chem.202500514

**Published:** 2025-04-07

**Authors:** Lilian S. Szych, Daniel S. Frost, Moritz J. Ernst, Jonas Bresien, Manuela Weber, Annelie Puhlmann, Luise Sander, Christian Müller

**Affiliations:** ^1^ Institute of Chemistry and Biochemistry Freie Universität Berlin Fabeckstraße 34/36 14195 Berlin Germany; ^2^ Institute of Chemistry Universität Rostock Albert‐Einstein‐Straße 3a 18059 Rostock Germany

**Keywords:** azaarsoles, cycloaddition, dichloroarsanes, heterocycles, main‐group chemistry

## Abstract

Easily accessible dichloroarsanes are suitable precursors for the synthesis of rarely explored triazaarsoles, the arsenic analogues of tetrazoles and triazaphospholes. Calculations on the DFT level gave insight into the electronic structure of such planar compounds, indicating a high degree of aromaticity. Our preliminary investigations pave the way for accessing and investigating a whole variety of new heterocycles that contain a low‐coordinated arsenic atom.

## Graphical abstract

Easily accessible dichloroarsanes are suitable precursors for the synthesis of novel triazaarsoles, the arsenic analogues of tetrazoles and triazaphospholes. Our findings pave the way to access and fully investigate such aromatic arsenic heterocycles in detail.

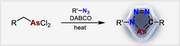

## 1 Introduction

**Scheme 1:**
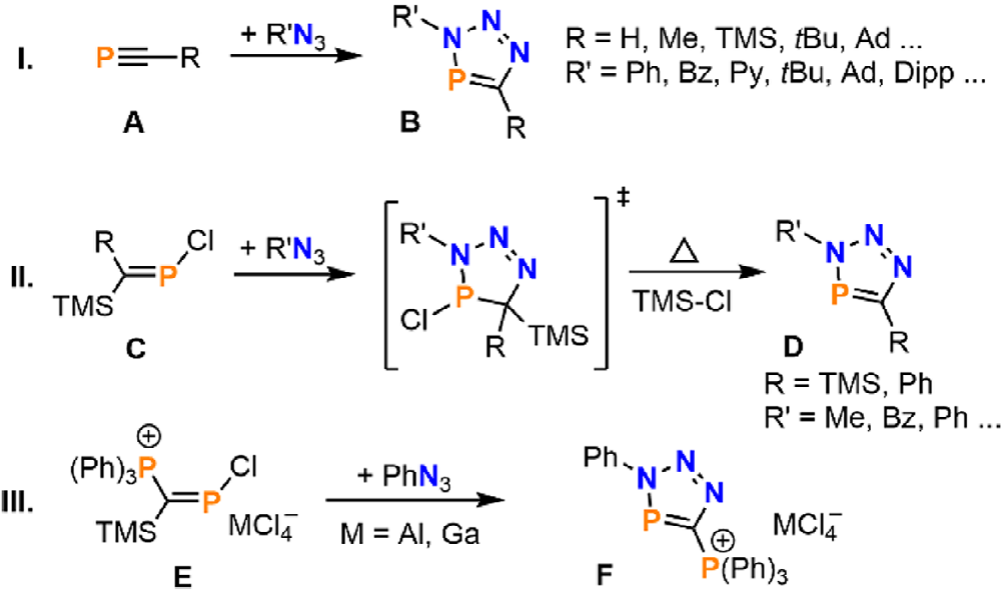
**I**. Synthesis of triazaphospholes **B** via the conversion of phosphaalkynes **A** with organic azides in cycloaddition reactions. **II**. Synthesis of triazaphospholes **D** by conversion of the chlorophosphaalkene **C** with organic azides via TMS–Cl elimination from a cyclic intermediate.^[^ ^5^ ^]^ **III**. Conversion of the 2‐triphenylphosphonium‐1‐chlorophosphaalkene **E** with phenyl azide, yielding the cationic 5‐phosphonium‐triazaphosphole **F**.^[^ ^6^ ^].^

The derivatives 3*H*‐1,2,3,4‐triazaphospholes are Hückel‐aromatic, five‐membered, and planar heterocycles that exhibit a low‐coordinated (*λ* ^3^ *σ* ^2^) phosphorus atom.^[^ ^1^, ^2^, ^3^ ^]^ As reported by Carrié and Regitz in 1984 independently for the first time, 3,5‐disubstituted triazaphospholes of the type **B** can be synthesized via the conversion of phosphaalkynes R–C≡P **A** with organic azides R–N_3_ in [3 + 2] cycloaddition reactions (Scheme 1, route I).^[^ ^4^, ^5^ ^]^

Unlike other “click‐reactions,” these conversions do not require a catalyst and are regioselective, due to the presence of a polarized P≡C triple bond.^[^ ^7^, ^8^, ^9^, ^10^, ^11^, ^12^ ^]^ The cycloaddition reactions of phosphaalkynes and azides were found to be highly tolerant to functional groups, allowing for the isolation of a vast variety of derivatives over the past years.^[^ ^8^, ^13^, ^14^, ^15^, ^16^ ^]^ As recently reported by us for the first time, cyaphido complexes of the type L_n_M–C≡P can also serve as dipolarophiles in cycloaddition reactions, since they readily react with a variety of azides.^[^ ^17^, ^18^, ^19^ ^]^ Recently, Goicoechea and Fernández conducted extensive computational mechanistic investigations using a model system on other factors influencing the 1,3 dipolar cycloaddition reactions such as regioselectivity and aromaticity.^[^ ^20^ ^]^ However, the availability and stability of the respective phosphaalkyne species (e.g., H‐C≡P,^[^ ^21^ ^]^ R─C≡P, R = Me,^[^ ^17^ ^]^ TMS,^[^ ^22^ ^]^ *t*Bu,^[^ ^23^, ^24^ ^]^ Mes,^[^ ^17^ ^]^ Mes* (= 2,4,6‐tri‐*tert*‐butylphenyl),^[^ ^17^ ^]^ Ter (= 2,6‐bis‐(2,4,6‐trimethylphenyl)‐phenyl))^[^ ^25^ ^]^ or cyaphido complexes^[^ ^17^, ^26^ ^]^ remains decisive for this synthetic route.

**Scheme 2:**
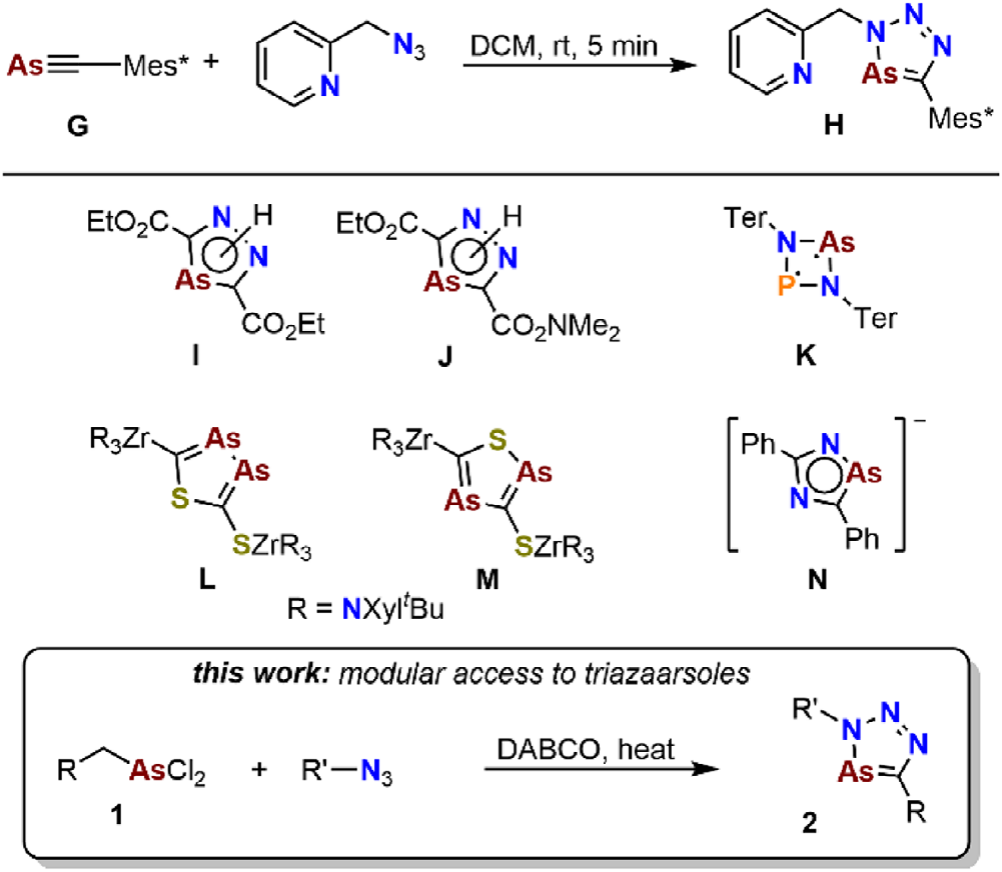
Synthesis of the triazaarsole **H** via conversion of the Mes*‐substituted arsaalkyne **G** with an azide (top) and examples of other reported arsenic heterocycles **I** to **N**.

Another, nowadays less widely used, synthetic approach toward triazaphospholes is the route described both by Carrié et al. and Märkl et al. This route involves the conversion of phosphaalkenes of type **C**, which are used as synthetic equivalents to phosphaalkynes. During the synthesis, instable cyclic intermediates aromatize via TMSCl elimination forming the respective triazaphospholes **D** (Scheme 1, route II).^[^ ^5^, ^27^ ^]^ Analogously, Schmidpeter and coworkers used the cationic phosphaalkene **E** as a synthon for the phosphoniumalkyne (([P≡C‐PPh_3_]^+^), which forms the cationic 5‐phosphonium‐3*H*‐1,2,3,4‐triazaphosphole **F** with phenyl azide (Scheme 1, route III).^[^ ^6^ ^]^ While triazaphospholes have been extensively investigated over the past years, their heavier analogues, triazaarsoles **H**, have rarely been investigated (Scheme 2).

Recently, our group reported on the conversion of Mes*‐C≡As **G** with an organic azide, yielding the first 3,5‐disubstituted triazaarsole derivative (Scheme 2). Hereby, again the polarization of the As≡C‐bond seems to be decisive for the observed regioselectivity.^[^ ^28^ ^]^ In contrast to phosphaalkynes as starting material, however, the heavier arsaalkynes are significantly more labile, which is due to the unfavorable overlapping of the As(4*p*)‐C(2*p*) orbitals. Until today, the Mes*‐substituted derivative Mes*‐C≡As remains the only reported stable arsaalkyne, which can be synthesized and isolated on a larger scale.^[^ ^29^ ^]^ Hence, the variation of the substitution pattern at the 5‐position of a triazaarsole ring systems is not possible via the route involving the conversion of arsaalkynes with azides.

Due to the strictly limited availability of triazaarsoles, investigations concerning the reactivity of those highly interesting species are limited to systems substituted by the Mes* substituent in α‐position to the arsenic atom. In fact, a structurally closely related diazaarsole was reported by Hamelin and coworkers, who generated arsaalkenes in situ, which were subsequently converted with ethyldiazoacetat or the Danishefsky‐diene to the respective arsanines **I** and **J**.^[^ ^30^ ^]^ Another structurally related species, a planar CN_3_As biradicaloid ring system **K,** was reported by Schulz et al. in 2016.^[^ ^31^ ^]^ Very recently, Weigend, Hohloch and co‐workers reported on the synthesis of arsenic heterocycles **L** and **M** using heavy cyanate anions as precursors.^[^ ^32^ ^]^ Moreover, Metha et al. reported on the synthesis of 1,2,4‐diazarsolide anions **N** by treating [K(DME)_x_]_3_[As_7_] with an azide.^[^ ^33^ ^]^

Consequently, future systematic investigations on the reactivity of triazaarsoles urgently necessitate the option to vary the substitution pattern at the C atom of the ring system. As previously reported, substituted phospha‐ or arsaalkenes can be used in cycloaddition reactions for the synthesis of diazaphospholes and diazaarsoles (vide supra). Characteristically, the alkene derivatives thereby exhibit β‐eliminable groups. Their elimination enables the formation of aromatic heterocycles. We now adapted this synthetic strategy to systematically synthesize triaazaarsoles and report here on a new synthetic approach to vary the substitution pattern of triazaarsoles at the C‐atom, starting from synthetically easily accessible acyclic alkyldichloroarsoles and organic azides (Scheme 2).

## 2 Results and Discussion

We anticipated that easily accessible alkyl dichloroarsanes of the type R–CH_2_AsCl_2_, respectively, R–CH(SiMe_3_)AsCl_2_ (**1**) might be suitable precursors for a base‐assisted, in situ generation of reactive, unsaturated species, which might react with organic azides R–N_3_ to the desired triazaarsoles. In this way, the introduction of sterically and electronically different substituents would be possible, e.g. introducing Ph, TMS, Mes, or even Tripp groups (Ph = phenyl; TMS = trimethylsilyl; Mes = mesityl; Tripp = (1,3,5‐tri(*iso*propyl)phenyl) at the C‐atom (5‐position). However, synthetic routes for the isolation of alkyl dichloroarsanes of the type R–CH_2_AsCl_2_ have been described much less frequently than routes toward their phosphorus analogues.^[^ ^34^, ^35^, ^36^, ^37^, ^38^, ^39^ ^]^ Depending on the nature of the substituent R, we started to synthesize the required precursor on different routes. In fact, we found that in cases where the required magnesium Grignard reagents react unselectively with AsCl_3_ (as observed for compounds **1c** and **1d**), alkyl zinc reagents turned out to be more suitable reagents for the selective conversion with AsCl_3_. All reported dichloroarsanes were characterized by means of ^1^H NMR and ^13^C{^1^H} NMR spectroscopy and, where possible, also by means of X‐ray crystallography.

**Scheme 3:**
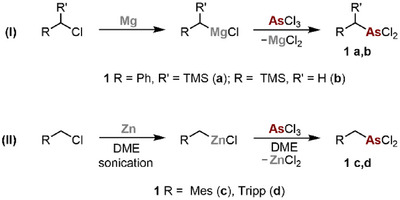
**(I)** Synthesis of compounds **1a** and **1b** via conversion of the corresponding magnesium Grignard reagents with AsCl_3_.^[^ ^40^, ^41^ ^]^ **(II)** Synthesis of compounds **1c** and **1d** via conversion of the corresponding organozinc reagents with AsCl_3_.

The dichloro(phenyl(trimethylsilyl)methyl)arsane (PhCH(SiMe_3_)AsCl_2_; **1a**) is a colorless oil, which was synthesized in a procedure analogous to the synthesis of Appel's dichlorophosphane using a magnesium Grignard reagent. In the last step, AsCl_3_ is used instead of PCl_3_, yielding the corresponding dichloroarsane **1a**.^[^ ^40^ ^]^ The dichloro((trimethylsilyl)methyl)arsane Me_3_SiCH_2_AsCl_2_ **1b** can be synthesized using a modified protocol by Wells et al.^[^ ^41^ ^]^ The Grignard reagent Me_3_SiCH_2_MgCl reacts with As(NEt_2_)_2_Cl at T = −80 °C and can be subsequently converted with HCl in Et_2_O to the desired product. The dichloro(2,4,6‐trimethylbenzyl)arsane MesCH_2_AsCl_2_ **1c** and the dichloro(2,4,6‐tri*iso*propylbenzyl)arsane TrippCH_2_AsCl_2_ **1d** were synthesized via conversion of the previously reported respective organozinc reagents of the type R─CH_2_ZnCl (**1c** R = Dipp, **1d** R = Tripp)^[^ ^17^ ^]^ with AsCl_3_ at T = 0 °C (Scheme 3).

The organozinc reagents must be handled at low temperatures to prevent the formation of side products.^[^ ^17^ ^]^ At room temperature, the isolated compound **1c** has a very viscous consistency, while compound **1d** is a slightly sticky solid. Compounds **1c** and **1d** crystallize at T = −20 °C yielding single crystals suitable for X‐ray analysis.^[^ ^42^ ^]^

**Figure 1:**
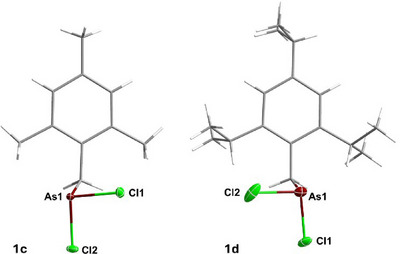
Molecular structures of **1c** (left) and **1d** (right) in the single crystal. Ellipsoids (for As and Cl) set at 50% probability. Color code: white = hydrogen, grey = carbon, green = chlorine, purple = arsenic. For compound **1d**, the nondisordered isomer is displayed. Selected bond lengths and angles: **1c**: As1–C1 1.9768(18) Å; As1–Cl1 2.1984(5) Å; As1–Cl2 2.1973(5) Å; **1d**: As1–C1 1.968(2) Å; As1–Cl1 2.1887(8) Å; As1–Cl2 2.1898(9) Å.

The molecular structures of **1c** and **1d** in the crystal are depicted in Figure 1 along with selected bond lengths and angles. Compound **1c** crystallizes in the space group *P*2_1_/*n*. The As–C bond (As1–C1 1.9768(18) Å) and the As–Cl bonds (As1–Cl1 2.1984(5) Å; As1–Cl2 2.1973(5) Å) in **1c** are in the range of the sum of the covalent radii of a corresponding single bond (∑*r*_cov_(As–C): 1.96 Å; ∑*r*_cov_(As–Cl): 2.20 Å).^[^ ^43^ ^]^ Compound **1d** crystallizes in the space group *P* $\bar{1}$. The unit cell contains two inequivalent moieties of **1d**, which differ in the orientation of their AsCl_2_ moieties. The bond lengths of both isomers are very similar and one of the isomers is disordered. In the nondisordered isomer, the As–C bond (As1–C1 1.968(2) Å) and the As–Cl bonds (As1–Cl1 2.1887(8) Å; As1–Cl2 2.1898(9) Å) in **1d** are, similar to compound **1c**, in the range of the sum of the covalent radii of a corresponding single bond.^[^ ^43^ ^]^

**Scheme 4:**
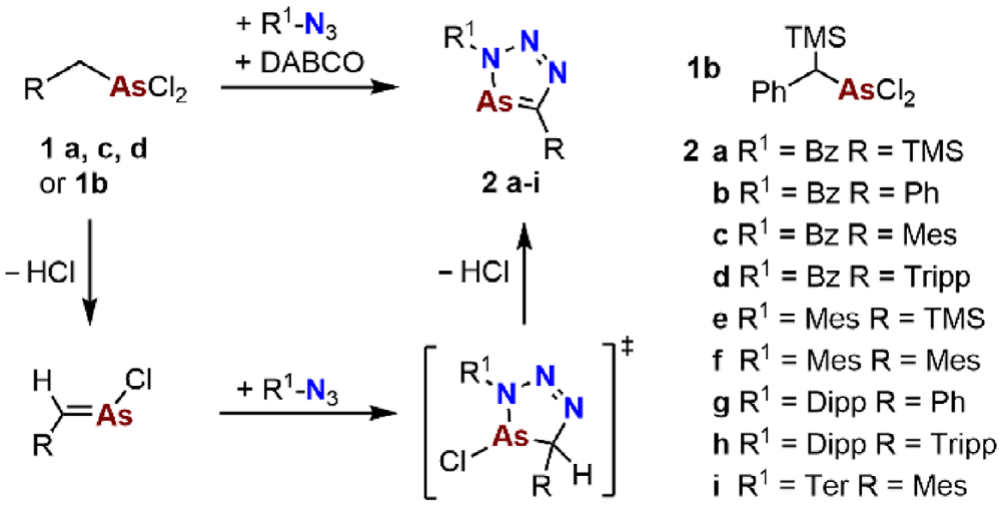
Synthesis via the proposed intermediate species, for synthesis of **2d**, **2g**, **2h** and **2i** addition of KI in situ, mechanism then proceeds via analogous iodo compounds. Please note that in case of **1b**, HCl, and TMSCl are eliminated in the reaction instead of two moieties of HCl.

Much to our delight, we found that the conversion of the dichloroarsoles Ph–CH(TMS)AsCl_2_ **1a** or R–CH_2_AsCl_2_ **1b‐d** with a variety of previously reported organic azides R–N_3_ (R = Bz,^[^ ^44^ ^]^ Mes,^[^ ^45^ ^]^ Dipp,^[^ ^46^ ^]^ Ter^[^ ^47^ ^]^) in the presence of DABCO (1,4‐diazabicyclo[2.2.2]octane) afforded indeed a variety of novel diazaarsoles (Scheme 4, **2a‐i**).

Dehydrochlorination reactions of functionalized alkyl dichlorophosphanes with DABCO have been reported previously.^[^ ^31^, ^48^, ^49^ ^]^ For example, Hamelin and co‐workers attempted to obtain functionalized phosphaalkenes from comparable dichlorophosphanes by dehydrochlorination with DABCO. However, it was not possible to isolate these species, and they had to be captured in situ (see Scheme 1, route II).^[^ ^31^ ^]^ Based on these observations and other reports on cycloaddition reactions with phosphaalkenes, a reaction mechanism can be postulated for the conversion of **1a‐d** to **2a‐i**. The in situ formed arsaalkene species forms a cyclic, short‐living intermediate and subsequently aromatizes by HCl elimination to the desired triazaarsoles **2a‐i** (Scheme 4). Thereby, the base DABCO supports the twofold deprotonation at the methylene bridge. However, neither the proposed cyclic intermediate nor the arsaalkenes could be detected spectroscopically. When monitoring the course of the reaction via ^1^H spectroscopy, only the reactants and/or the products were identified NMR spectroscopically. Particularly the signal of the methylene bridge of the arsenic precursors R–C*H*_2_AsCl_2_ is a suitable probe to follow the reaction, as it appears in the range of δ(ppm) = 2.1–3.9. Thus, full conversion to the desired triazaarsole species is achieved when this signal has fully disappeared in the ^1^H NMR spectrum of the reaction solution. The ideal reaction conditions (temperature, time) were found to be dependent on the steric and electronic nature of the dichloroarsoles R–CH_2_AsCl_2_. Whereas, for smaller substituents, we found that milder conditions (lower temperatures and shorter reaction times) are suitable, sterically more demanding substrates require more harsh reaction conditions to achieve a complete substrate conversion. The polar solvents Et_2_O or DME were found to give the best results, whereby DME was favored regarding its significantly higher boiling point (Table 1).

**Table 1:** Reaction conditions and yields of the triazaarsole synthesis.

Compound	R^1^	R	Conditions	Yield [%]
**2a**	Bz	TMS	DME, 80 °C, 3 h	25
**2b**	Bz	Ph	Et_2_O, RT, 3 d	88
**2c**	Bz	Mes	DME, 85 °C, 50 h	45
**2d**	Bz	Tripp	DME, 80 °C, 48 h^[^ ^a^ ^]^	41
**2e**	Mes	TMS	DME, 85 °C, 1 d	66
**2f**	Mes	Mes	DME, 85 °C, 50 h	64
**2g**	Dipp	Ph	DME, 85 °C, 6 d^[a]^	46
**2h**	Dipp	Tripp	DME, 85 °C, 2 d^[a]^	42
**2i**	Ter	Mes	DME, 80 °C, 2 d^[a]^	36

^[a]^Procedure using KI in situ.

Depending on the stability and solubility of the triaazaarsole products, the workup procedure was varied (see Supporting Infomation for experimental details). Interestingly, we found that for the reaction of sterically demanding combinations involving starting materials bearing Dipp/Tripp/Ter substituents (**2d**, **2** **g**, **2** **h**, **2i**), a halogen exchange can be performed in situ. This is achieved by adding 2 eq of KI to a mixture of the respective dichloroarsole, azide, and DABCO. The formation of the iodo species in situ is indicated by a color change of the reaction solutions to orange during heating.

Please note that we have also tried combining further azide/chloroarsane combinations. However, in some cases, we were unfortunately not successful to separate the products from side products (See Supporting Infomation).

### 2.1 Structural Characterization of Triazaarsoles

**Figure 2:**
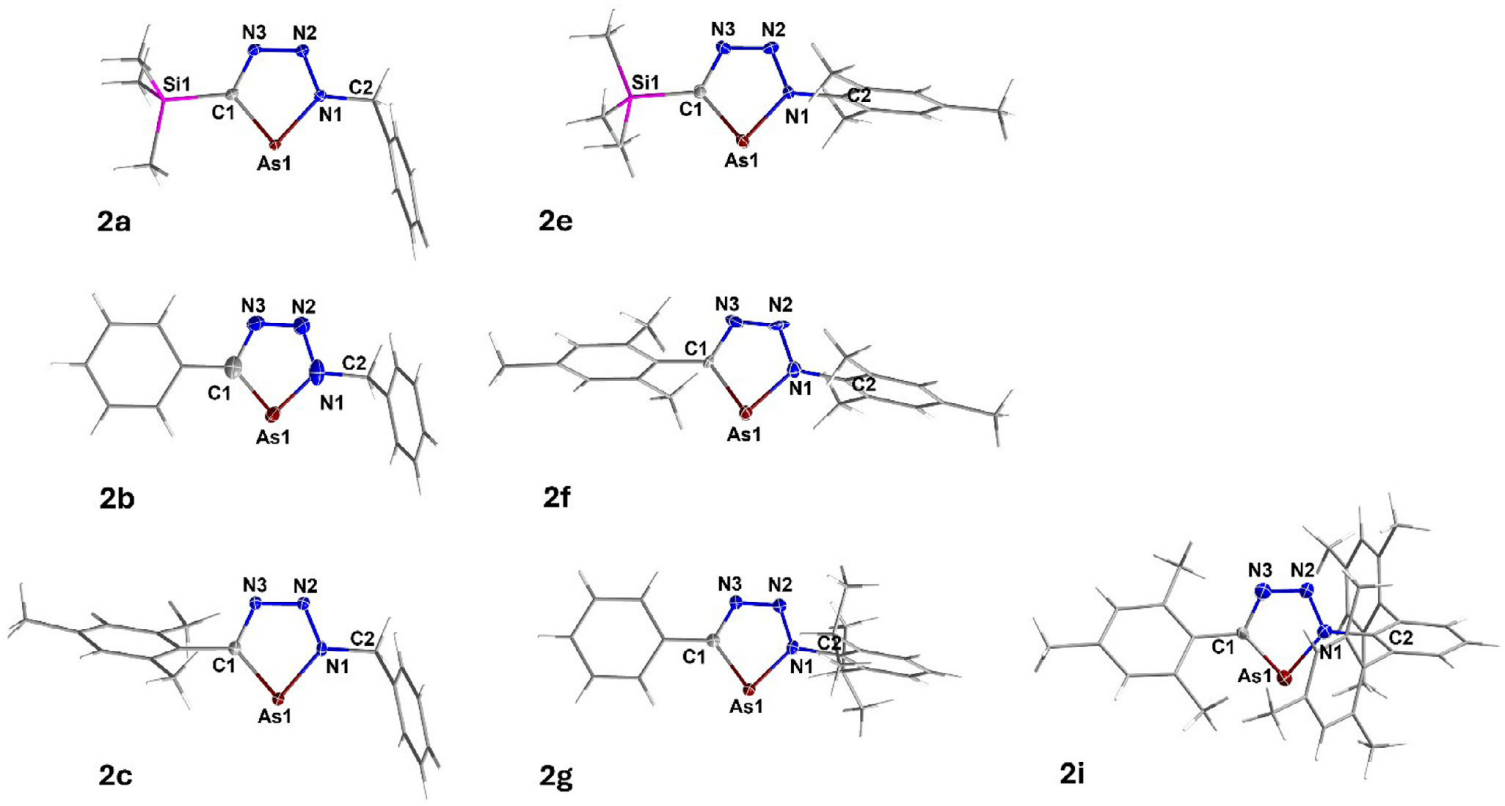
Molecular structures of **2a‐c, 2e‐g,** and **2i** in the single crystal. Ellipsoids (for As1, N1, N2, N3, and C1) set at 50% probability. Color code: white = hydrogen, grey = carbon, blue = nitrogen, purple = arsenic. Eventually, co‐crystallized solvent molecules are omitted for clarity. Structure **2b** is disordered along the positions of the N atoms and As atom, displayed is the major component (70%). Structure **2c** contains two inequivalent moieties per unit cell, which slightly differ with respect to orientation of Bz substituent to CAsN_3_ ring plane. Only one moiety is displayed. Structure **2g** contains two inequivalent moieties per unit cell, one of them is disordered with respect to the N/As atoms. Displayed is the nondisordered isomer.

Single crystals, suitable for X‐ray diffraction, were obtained for compounds **2a‐c**, **2e‐g,** and **2i** (for details see SI). All crystallographically characterized compounds exhibit planar, five‐membered C‐As–N_3_ ring systems, whereby also both the R–C atom and the R─N1 atom exhibit a planar geometry (Figure 2). The C1─As and the As─N1 bonds (see Table 2), as well as the other bonds within the five‐membered arsenic heterocycle all lie in‐between what would be expected for corresponding single and double bonds, indicating a significant degree of π‐conjugation and aromaticity (for aromaticity: see calculations, vide infra). The unit cell of **2g** contains two inequivalent moieties per unit cell, one of them disordered (N and As positions in ring system). In both systems, the Ph ring lies in one plane with the AsCN_3_ heterocycle, and the ring system of the Dipp substituent is shielding the AsCN_3_ moiety orthogonally. Each one of the ^Dipp^ *i*Pr groups is located above and below the AsCN_3_ plane, whereby the CH(CH_3_)_2_ atoms are facing toward AsCN_3_ system. Interestingly, the bond parameters of both isomers differ significantly (see Table 2, displayed for the disordered isomer is only component (50%) since the molecular parameters for the other component are very similar). In **2i**, the central phenyl ring of the Mes substituent (at the C atom) and the Ter substituent (at the N atom) are twisted and are not in one plane with the AsCN_3_ heterocycle, as also observed for compound **2g**. Analogously to the ^Dipp^ *i*Pr groups in**2g**, the ^Ter^Mes substituents in **2i** are located above and below the AsCN_3_ moiety.

**Table 2:** Structural parameters of triazaarsoles.

Compound	R^1^/R	C–As [Å]	As–N1 [Å]	C1‐As1‐N1 [°]
**2a**	Bz/TMS	1.8367 (19)	1.8278 (18)	82.72 (8)
**2b**	Bz/Ph	1.765 (3)	1.745 (3)	86.68 (12)
**2c**	Bz/Mes^[^ ^a^ ^]^	1.842 (3) 1.843 (3)	1.834 (2) 1.832 (2)	81.62 (11) 81.53 (11)
**2e**	Mes/TMS	1.8333 (18)	1.8208 (15)	83.16 (7)
**2f**	Mes/Mes	1.827 (3)	1.826 (3)	82.34 (12)
**2g**	Dipp/Ph^[^ ^b^ ^]^	1.850 (2) 1.793 (2)	1.8291 (17) 1.7413 (18)	81.40 (8) 85.42 (10)
**2i**	Ter/Mes	1.824 (5)	1.818 (4)	83.29 (18)

^[a]^Structure **2c** contains two inequivalent moieties per unit cell, which slightly differ with respect to orientation of Bz substituent to CAsN_3_ ring plane, both isomers are indicated in the table.

^[b]^Structure **2g** contains two inequivalent moieties per unit cell, one of them is disordered with respect to the N/As atoms. Displayed are parameters of the nondisordered isomer and the major component of the disordered isomer.

### 2.2 Computational Results

Previous calculations on the parent triazapnictoles PnN(H)N_2_C(H) (Pn = N, P, As) showed that the HOMO‐LUMO gap becomes smaller going from the tetrazole to the triazaarsole. Both the HOMO and LUMO of the triazaphosphole and the triazaarsole show large coefficients with π‐symmetry at the P/As atom. The tetrazole also exhibits a coefficient with π‐symmetry at the N atom, which is much smaller than the heavier analogues. The lone pair at the pnictogen atom is in all cases reflected by an energetically low‐lying HOMO−2.^[^ ^28^ ^]^

**Figure 3:**
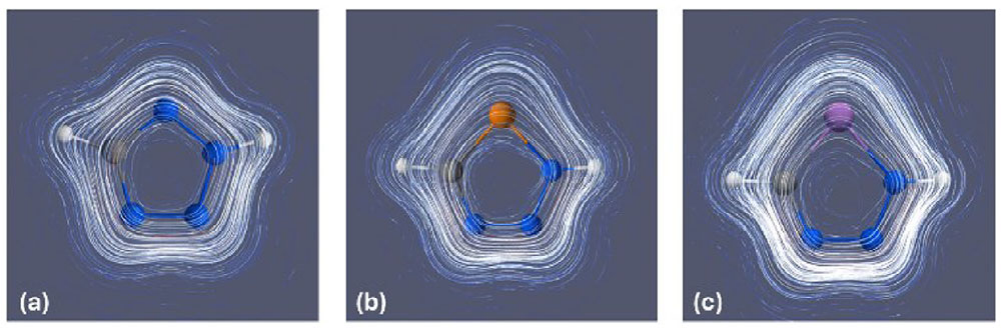
Streamline plot of the induced ring current densities^[^ ^47^ ^]^ of a) NN(H)N_2_C(H), (b) PN(H)N_2_C(H), and (c) AsN(H)N_2_C(H). Colour code: white = hydrogen, grey = carbon, blue = nitrogen, orange = phosphorus, purple = arsenic.

We were further interested in investigating the properties of the π‐systems and the resulting aromaticity of triazaarsoles. Some studies on triazaphosphole derivatives have been reported in the past, indicting significant aromatic character for these compounds.^[^ ^8^, ^50^ ^]^ The aromaticity of the parent triazapnictoles of the type PnN(H)N_2_C(H) (Pn = N, P, As) will be discussed in the following by evaluating molecular parameters such as the magnetically induced ring current^[^ ^51^, ^52^ ^]^ and Nucleus Independent Chemical Shift (NICS) values (for computational details, see Supporting Infomation).^[^ ^53^, ^54^, ^55^ ^]^ As typically observed for aromatic compounds, all investigated triazapnictoles PnN(H)N_2_C(H) (Pn = N, P, As) exhibit a diatropic ring current surrounding the heterocycles above and below the ring plane (Figure 3).

The net induced ring current was derived by integration of the current density along the vertical ring sections to the respective ring center (Table 3).^[^ ^51^, ^52^, ^56^, ^57^ ^]^ From the calculations, it becomes obvious that the computed ring currents are similar for all three derivatives (>13 nA/T). Furthermore, the ring currents are positive for all compounds, which implies a net diatropic current. The determined NICS(0) values between − 14.7 and − 16.1 and NICS(1)*_zz_* values between − 37.1 and − 38.5 further indicate the aromaticity of the corresponding heterocycles.

**Table 3:** Net Induced Currents, NICS(0) and NICS(1)*_zz_* values of **Pn**N(H)N_2_C(H) (Pn = N, P, As).^[^ ^49^, ^50^, ^51^ ^]^

	Induced ring current [nA/T]	NICS(0) [ppm]	NICS(1)_zz_ [ppm]
**N**N(H)N_2_C(H)	13.8	−14.7	−38.5
**P**N(H)N_2_C(H)	14.1	−15.6	−37.9
**As**N(H)N_2_C(H)	14.2	−16.1	−37.1

## 3 Conclusion

A new synthetic approach to various novel triazaarsoles was presented, starting from easily accessible dichloroarsanes. The here reported synthesis of triazaarsoles is expected to proceed via a reactive intermediate, that is generated in situ and undergoes a [3 + 2] cycloaddition reactions with organic azides. Using this route, the variation of the C‐substituent at the 5‐position of triazaarsoles is possible, allowing even for the introduction of larger, sterically rather demanding substituents. Thus, the availability of triazaarsoles is no longer restricted to the stability and isolation of the respective arsaalkynes. This way, we were able to synthesize a total of nine new triazaarsoles, that contain different substituents, both at the nitrogen atom and the carbon atom, that is adjacent to the arsenic atom. All compounds were characterized by means of NMR spectroscopy and mass spectrometry and, in most cases, also crystallographically. Calculations on the net‐induced ring current and on NICS values indicate that the parent triazaarsole AsN(H)N_2_C(H) is an aromatic compound. Further studies on the novel triazaarsoles, such as the investigation of their coordination chemistry and their further functionalization, are currently carried out in our laboratories.

## Supporting Information

The authors have cited additional references within the Supporting Information.^[^ ^58^, ^59^, ^60^, ^61^, ^62^, ^63^, ^64^, ^65^, ^66^, ^67^, ^68^, ^69^, ^70^, ^71^, ^72^, ^73^, ^74^, ^75^, ^76^, ^77^, ^78^, ^79^, ^80^, ^81^, ^82^, ^83^, ^84^, ^85^, ^86^, ^87^, ^88^, ^89^, ^90^, ^91^, ^92^, ^93^, ^94^, ^95^, ^96^ ^]^ The Supporting Information includes detailed syntheses for all of the reported compounds, analytical, and spectroscopic data and a summary of the computational techniques used and relevant results.

## Conflict of Interests

The authors declare no conflict of interest.

## Supporting information



Supporting Information
