# Supplementary material for: On the Facile Synthesis of Triazaarsoles Using Acyclic Precursors
Source: Chemistry. 2025 Apr 7;31(27):e202500514. doi: 10.1002/chem.202500514 (PMC12080306; doi:10.1002/chem.202500514)
Supplement: Supplementary file 1 — Supporting Information [file CHEM-31-e202500514-s001.pdf]

Supporting Information

This file includes:

1 Experimental 2

2 Syntheses of compounds 4

3 Computational Details 22

4 Structure elucidation 25

5 References 34

1 Experimental

General information. If not stated otherwise, all reactions and product manipulations were carried out using standard Schlenk-line techniques under an inert atmosphere of argon, or in an argon filled glovebox (MBraun UNIlab glovebox maintained at <0.1 ppm H_2O and <0.1 ppm O_2). All glassware was flame-dried and cooled under argon atmosphere. Solvents were transferred using syringes or teflon cannula, which were purged with argon prior to use. *N*-pentane (Sigma Aldrich HPLC grade) and dichloromethane (VWR HPLC grade) were purified using an MBraun SPS-800 solvent system and degassed. 1,2-Dimethoxyethane DME (Sigma Aldrich HPLC grade) and diethyl ether Et_2O (Sigma Aldrich HPLC grade) were distilled over potassium/benzophenone. Diethylenglycoldimethylether (Diglyme, Sigma Aldrich) was dried over activated 4 Å molecular sieves.

C_6D_6 (Sigma Aldrich, 99.5%), $CDCl_3$ (Sigma Aldrich, 99.5%) and DCM-d_2 (Sigma Aldrich, 99.5%) were degassed, dried over KH and subsequently condensed for purification. All dry solvents were stored under argon in gas-tight Schlenk flasks over activated 4 Å molecular sieves. If working under non-inert conditions (indicated in the protocols if applicable), then the solvents mentioned above were used as received without further purification/drying. Ethyl acetate EtOAc (Sigma Aldrich), *n*-hexane (Sigma Aldrich HPLC grade) and $MgSO_4$ (Sigma Aldrich) were used as received for non-inert workup. Reactants were either obtained from commercial sources or synthesized as detailed in Table S1.

Table S1: Origin and purification of solvents and reactants.

**Substance**	**Origin**	**Purification**
$TMSCH_2Cl$	Sigma Aldrich	used as received
$MesCH_2Cl$	synthesized[14]	used as synthesized
$TrippCH_2Cl$	synthesized[14]	used as synthesized
Zn dust 10 µm	Aldrich	used as received
$AsCl_3$	old stock	dried over mol sieves, condensed, degassed (freeze-pump-thaw)
BzN_3	synthesized[40]	used as synthesized

Substance	Origin	Purification
$MesN_3$	synthesized[45]	used as synthesized
$DippN_3$ (2,6-di*iso*-propyl-1-azido-benzene)	synthesized[46]	used as synthesized
$TerN_3$ (2,6-dimesityl-phenyl azide)	synthesized[47]	used as synthesized
Mg turnings	Roth	used as received
$As(NEt_2)_2Cl$	synthesized[58]	used as synthesized
α-chlorobenzyl trimethylsilan	synthesized[59]	used as synthesized
HCl in Et_2O 2 M	ThermoFisher	used as received
DABCO	Sigma Aldrich	sublimed
KI	Sigma Aldrich	dried at 100 °C *in vacuo* for 2 days

Please note: In the manuscript/SI, RT (room temperature) = “ambient temperature” refers to 25(2) °C.

NMR spectra. The NMR samples were prepared inside an inert atmosphere glovebox (N_2 atmosphere, see general information) in Young NMR tubes fitted with a gas-tight valve; or prepared in a Schlenk glassware using a constant Argon flow and a suba seal. NMR spectra were acquired on either Bruker (AVANCE 500) or JEOL (ECX 400, Lamba 400, ECP 500, ECZ 600) NMR spectrometers. ^{1}H and ^{13}C NMR spectra were referenced to their respective solvent resonance (^{1}H NMR C_6D_6: δ = 7.16 ppm, DCM-*d*2: 3.58 ppm, $CDCl_3$: 7.26 ppm; ^{13}C NMR C_6D_6: δ = 128.4 ppm, DCM-*d*2: 53.8 ppm, $CDCl_3$: 77.2 ppm). Multiplicities are defined as (s) for singlets, (d) for doublets, (t) for triplets, (quin) for quintets, sept for septets, (m) for multiplets, and the addition of (br.) for a broad signal.

Mass spectrometry. High resolution mass spectrometry (HRMS) was performed on an *Agilent 6210 ESI-TOF*.

Sonication bath. For activation of Zn dust, a sonication bath of used, type: ELMA Transsonic 310/H.

2 Syntheses of compounds

2.1 Synthesis of chloroarsane precursors

2.1.3 Dichloro(phenyl(trimethylsilyl)methyl)arsane (1a)

The synthesis is carried out analogously as previously described for the P analogue.[40] To a suspension of Mg turnings (0.83 g, 34 mmol, 1.1 eq.) in Et_2O (35 ml), α-chlorobenzyl trimethylsilan (6.15 g, 30.9 mmol, 1.0 eq.) is slowly added. The resulting reaction mixture is refluxed for 1 h at 40 °C, whereupon the mixture darkens in color. Afterwards, the mixture is filtered (cannula filter) and added dropwise at 0 °C over a period of 1 h to a solution of $AsCl_3$ (5.6 g, 30.9 mmol, 1.0 eq.) in Et_2O (15 ml). After stirring the resulting mixture for 1 h at 0 °C, the resulting suspension is filtered, and all volatile components are removed *in vacuo* at 0 °C (1 x 10^{-3} mbar). The resulting turbid and darkish product is condensed *in vacuo* at 90 °C (1 x 10^{-3} mbar) and the desired pure product **1a** is isolated in form of a colorless liquid (4.21 g, 13.9 mmol, 45%).

M($C_{10}H_{15}SiAsCl_2$) = 309.14 g/mol. **^{1}H NMR** (DCM-*d*$_2$, 400.53 MHz, 293.45 K) *δ* [ppm] = 7.32 – 7.40 (m, 2H, Ar*H*); 7.20 – 7.30 (m, 1H, Ar*H*); 7.10 – 7.15 (m, 2H, Ar*H*); 3.42 (s, 1H, C*H*); 0.22 (s, 9H, $SiCH_3$). **^{13}C{^{1}H} DEPT 135 NMR** (DCM-*d*$_2$, 100.71 MHz, 294.95 K) *δ* [ppm] = 137.4 (s, Ar*C*); 129.7 (s, Ar*C*H); 129.3 (s, Ar*C*H); 127.0 (s, Ar*C*H); 55.4 (s, *C*H); −0.5 (s, Si*C*H_3).

Figure S1: NMR spectra of **1a** (DCM-*d*$_2$ solvent signals (^{1}H NMR: 5.32 ppm, ^{13}C NMR: 54.0 ppm) and grease signals (^{1}H NMR: 0.08 ppm, ^{13}C NMR: 1.3 ppm) indicated by asterisks).

^{1}H NMR spectrum

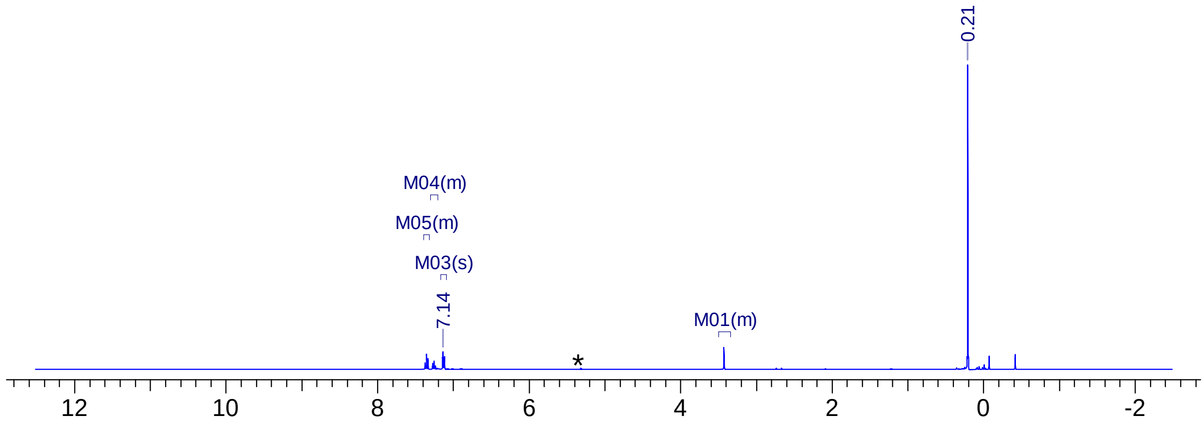

^{13}C DEPT 135 NMR spectrum

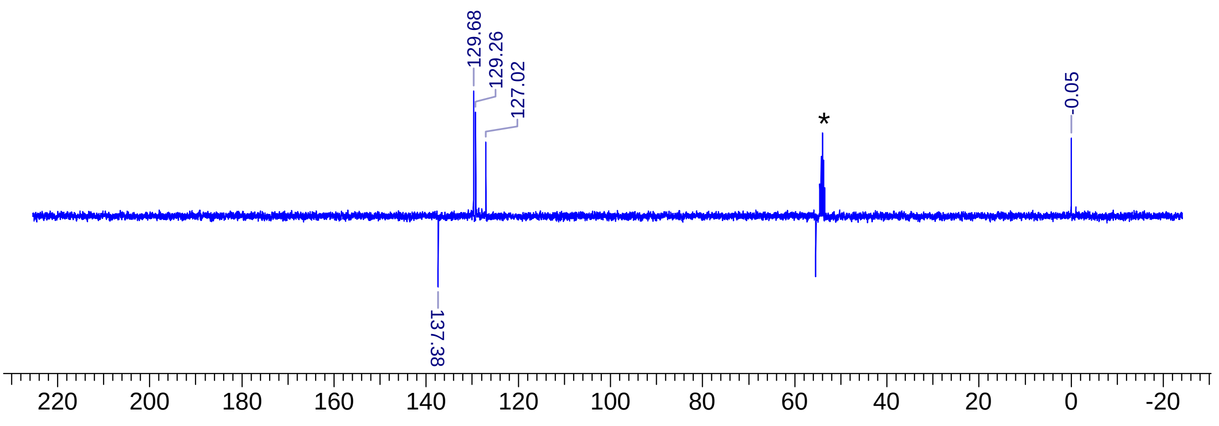

2.1.2 Dichloro((trimethylsilyl)methyl)arsane (1b)

The compound is synthesized according to a modified literature procure from Wells *et al*.[41] Mg turnings (1.46 g, 60 mmol, 1.36 eq.) are activated by stirring for 12 hours under an argon atmosphere. Et_2O (60 ml) is added and a solution of (chloromethyl)trimethylsilane Me_3SiCH_2Cl (5.4 g, 44 mmol, 1.0 eq) in Et_2O (20 ml) is added dropwise. The reaction mixture is refluxed for 15 h at 40 °C with a reflux condenser. The resulting suspension is filtered *via* a celite-packed frit yielding a clear filtrate. This filtrate is slowly added at −80 °C to $As(NEt_2)_2Cl$ (11.3 g, 44 mol, 1.0 eq) in Et_2O (50 ml) and stirred for 12 h at room temperature. Additional Et_2O (200 ml) is added to the reaction mixture and a solution of HCl in Et_2O (2.0 M, 66 ml, 132 mmol, excess) is added and the resulting solution is stirred for 12 hours at room temperature. The reaction mixture is filtered *via* a glass frit (G4). From the resulting clear solution all volatile components are removed *in vacuo*, yielding a colorless oil. The desired product **1b** is obtained by distillation at 35 °C (3.6 x 10^{-2} mbar to 8.1 x 10^{-3} mbar) in form of a colorless solution (8.04 g, 34.7 mmol, 79%). ^{1}H NMR spectroscopy and comparison to the reported literature of the phosphorus analogue confirms the formation of the product.[60]

M($C_4H_{11}SiAsCl_2$) = 233.04 g/mol. **^{1}H NMR** (DCM-*d*$_2$, 400.53 MHz, 292.85) δ [ppm] = 2.12 (s, 2H, C*H*$_2$); 0.21 (s, 9H, SiC*H*$_3$). **^{13}C{^{1}H} NMR** (DCM-*d*$_2$, 100.51 MHz, 293.75 K) δ [ppm] = 43.1 (s, *t*Bu*C*); 0.6 (s, TMS-*C*H$_3$); 27.9 (s, *C*H$_2$).

Figure S2: NMR and IR spectra of **1b** (DCM-d_2 solvent signals (^{1}H NMR: 5.32 ppm, ^{13}C NMR: 54.0 ppm) and grease signals (^{1}H NMR: 0.08 ppm, ^{13}C NMR: 1.3 ppm) and Et_2O signals (^{1}H NMR: 3.48 ppm, 3.65 ppm, ^{13}C NMR: 15.3 ppm) indicated by asterisks).

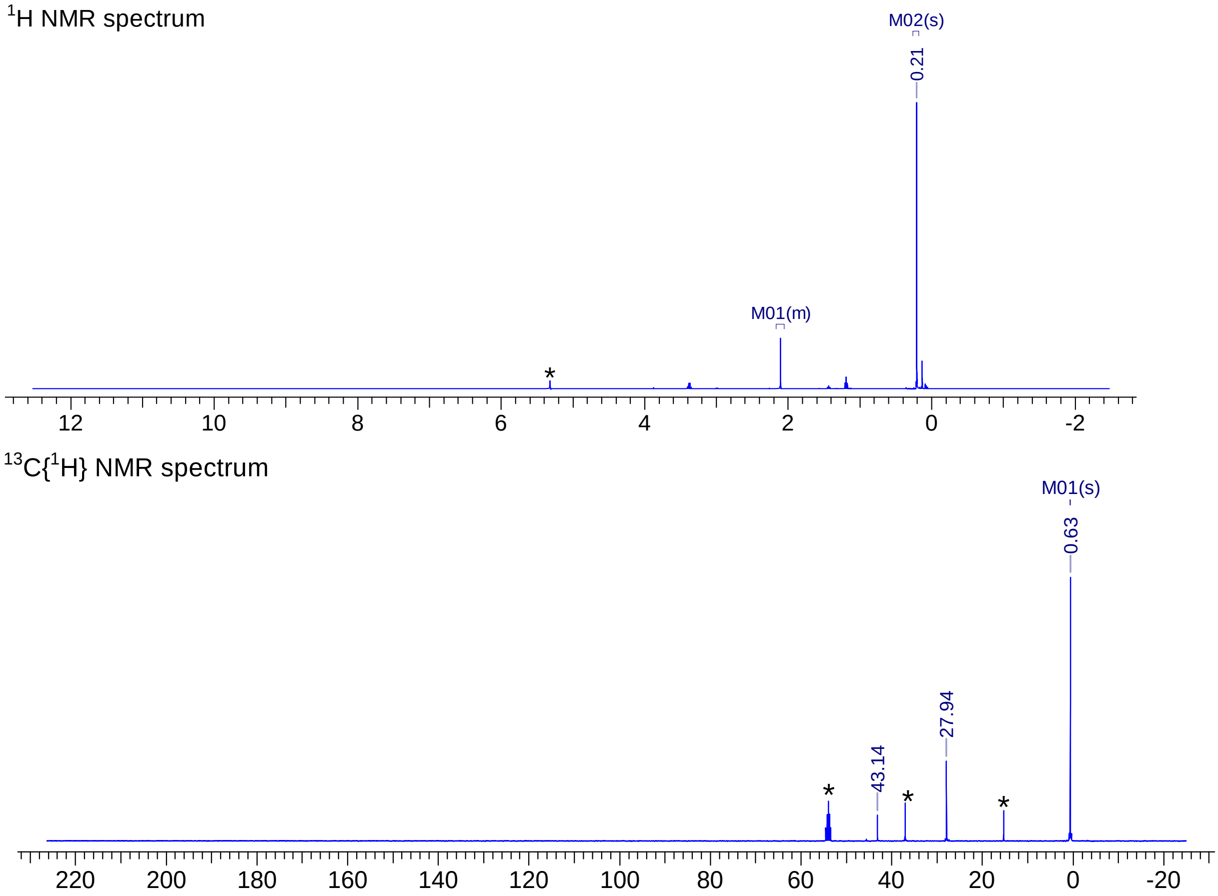

2.1.4 Dichloro(2,4,6-trimethylbenzyl)arsane (1c)

Zn dust (size 10 µm, 9.85 g, 150.6 mmol, 1.98 eq.) is dispersed in DME (20 ml) and activated in a sonication bath for 3 hours. Afterwards, a solution of 1,3,5-trimethylbenzyl chloride (12.8 g, 75.9 mmol, 1 eq.) in DME (60 ml) is added dropwise over a period of 20 min at 10 °C to the reaction solution. Subsequently, the reaction mixture is stirred at 10 °C for another 2 h and then filtered (cannula filter), yielding a yellowish filtrate. In another flask, $AsCl_3$ (15.0 g, 6.20 ml, 82.7 mmol, 1.09 eq.) is added to DME (30 ml), the resulting mixture is cooled to 0 °C and the filtrate is then added dropwise over a period of 20 min. The reaction mixture is stirred for another hour at 0 °C and subsequently warmed to room temperature overnight. All volatile components are removed *in vacuo* (1 x 10^{-3} mbar) yielding a colorless oil. DCM (40 ml) is added, and the resulting turbid suspension is stirred for 10 min at room temperature and then filtered (cannula filter). All volatile components are removed *in vacuo* (1 x 10^{-3} mbar), yielding the desired product in form of a colorless oil. The product **1c** is stored in the

dark at −30 °C, whereupon it solidifies to yield crystals suitable for Xray analysis. Yield: 19.1 g (68.4 mmol, 90.1%). Often, the product contains residue DME, the amount can be estimated *via* ^{1}H NMR spectroscopy and the DME concentration can be taken into account when calculating the amount of material in subsequent reactions.

M($C_{10}H_{13}AsCl_2$) = 279.02 g/mol. **^{1}H NMR** (C_6D_6, 300.37 MHz, 290.95 K) δ [ppm] = 6.64 (s, 2H, Ar*H*); 3.67 (s, 2H, C*H*$_2$); 2.05 – 2.07 (m, 9H, Mes-C*H*$_3$). **^{13}C NMR** (C_6D_6, 100.42 MHz, 290.85 K) δ [ppm] = 137.8 (s, Ar*C*); 137.2 (s, Ar*C*); 137.1 (s, Ar*C*); 130.2 (s, Ar*C*H); 129.8 (s, Ar*C*H); 127.5 (s, Ar*C*H); 47.4 (s, *C*H$_2$); 21.5 (m, Mes-*C*H$_3$); 19.5 (s, Mes-*C*H$_3$). **ESI-TOF (m/z):** 279.95 g/mol ($C_{10}H_{14}AsCl_2^+$ = [M+H$^+$]).

Figure S3: NMR and IR spectra of **1c** (C_6D_6 solvent signals (^{1}H NMR: 7.16 ppm, ^{13}C NMR: 128.4 ppm) and grease signals (^{1}H NMR: 0.08 ppm, ^{13}C NMR: 1.3 ppm) and DME signals (residue in this sample approx. 30%, ^{1}H NMR: 3.10 ppm, 3.27 ppm, ^{13}C NMR: 59.0 ppm, 72.3 ppm) indicated by asterisks).

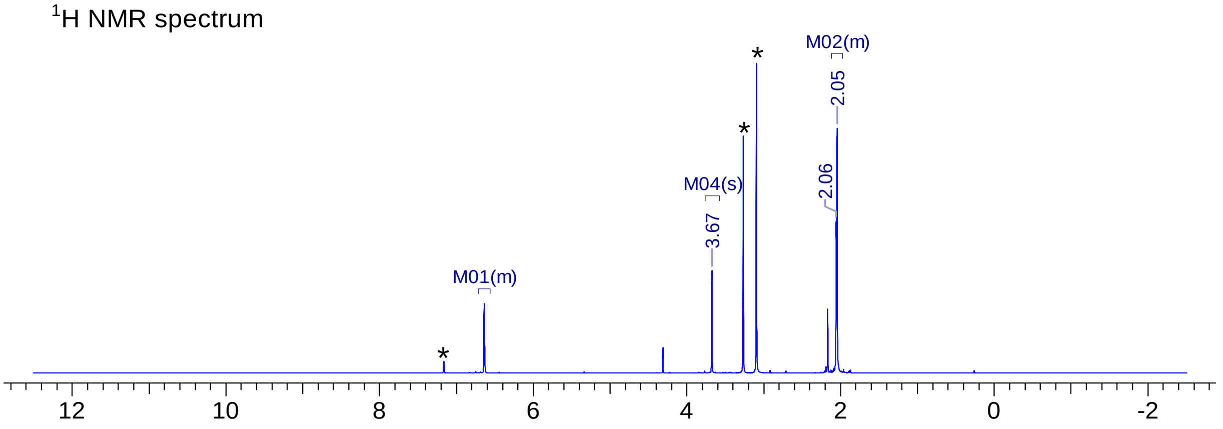

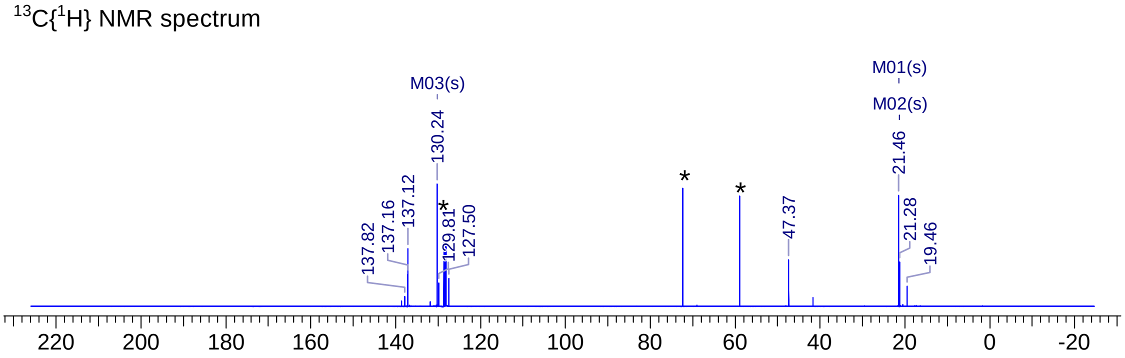

2.1.5 Dichloro(2,4,6-triisopropylbenzyl)arsane (1d)

Zn dust (size 10 µm, 5.50 g, 84.0 mmol, 4.42 eq.) is dispersed in DME (20 ml) and activated in a sonication bath for 7 hours. Afterwards, a solution of 1,3,5-tri*iso*propylbenzylchlorid (4.80 g, 19.0 mmol, 1 eq.) in DME (60 ml) is added and the reaction mixture is stirred for one day at 75 °C. Subsequently, the reaction mixture is cooled to room temperature and filtered (cannula filter), yielding a yellowish solution. In another flask, $AsCl_3$ (10.3 g, 4.80 ml, 56.9 mmol, 3 eq.) is added to DME (10 ml), the resulting mixture is cooled to 0 °C and the filtrate was added dropwise over a period of 20 min. The reaction mixture is stirred for another 1 h at 0 °C and subsequently warmed to room temperature overnight. All volatile components are removed *in vacuo* (1×10^{-3} mbar), resulting in a colorless oil. DCM (40 ml) is added, and the resulting turbid suspension is stirred for 10 min at room temperature and then filtered (cannula filter). All volatile components are removed *in vacuo* (1×10^{-3} mbar), yielding a sticky, colorless solid which fully solidifies upon storage in the fridge overnight. The product **1d** is stored in the dark at −30 °C. Yield: 5.35 g (14.7 mmol, 77.4%).

M($C_{16}H_{25}AsCl_2$) = 363.18 g/mol. **^{1}H NMR** (C_6D_6, 399.37 MHz, 290.65 K) δ [ppm] = 7.06 (s, 2H, Ar*H*); 3.94 (s, 2H, C*H*$_2$); 3.08 (hept, *J* = 6.9 Hz, 2H, *i*PrC*H*); 2.27 (hept, *J* = 6.9 Hz, 1H, *i*PrC*H*); 1.16 (d, *J* = 6.9 Hz, 6H, *i*PrC*H*$_3$); 1.12 (d, *J* = 6.9 Hz, 12H, *i*PrC*H*$_3$). **^{13}C NMR** (C_6D_6, 100.42 MHz, 290.65 K) δ [ppm] = 148.9 (Ar*C*); 148.3 (s, Ar*C*); 124.5 (s, Ar*C*H); 122.3 (s, Ar*C*H); 45.2 (s, *C*H$_2$); 35.0 (s, *i*Pr*C*H); 31.2 (s, *i*Pr*C*H); 24.7 (s, *i*Pr*C*H$_3$); 24.5 (s, *i*Pr*C*H$_3$). **ESI-TOF (m/z):** 363.98 g/mol ($C_{16}H_{26}AsCl_2^+$ = [M+H$^+$]).

Figure S4: NMR spectra of **1d** (C_6D_6 solvent signals (^{1}H NMR: 7.16 ppm, ^{13}C NMR: 128.4 ppm) indicated by asterisks).

^{1}H NMR spectrum

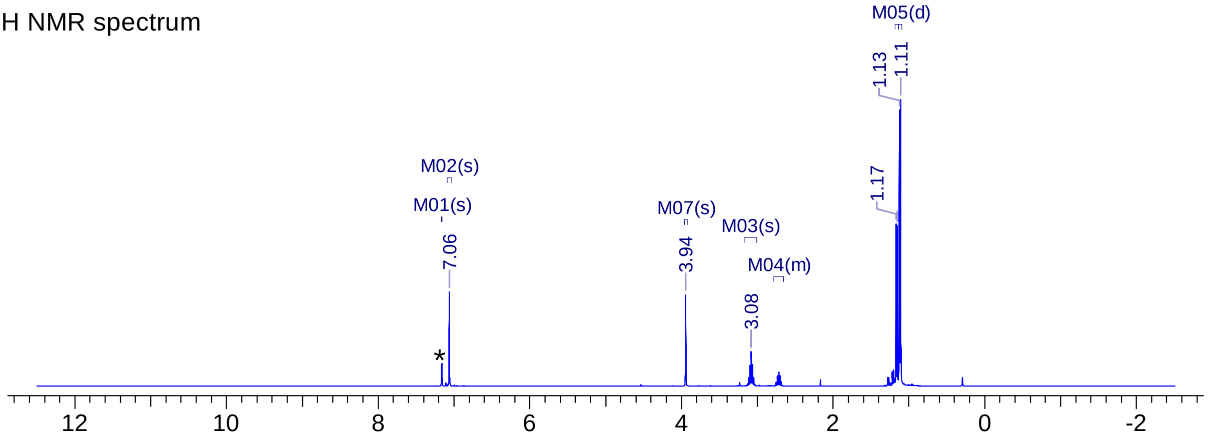

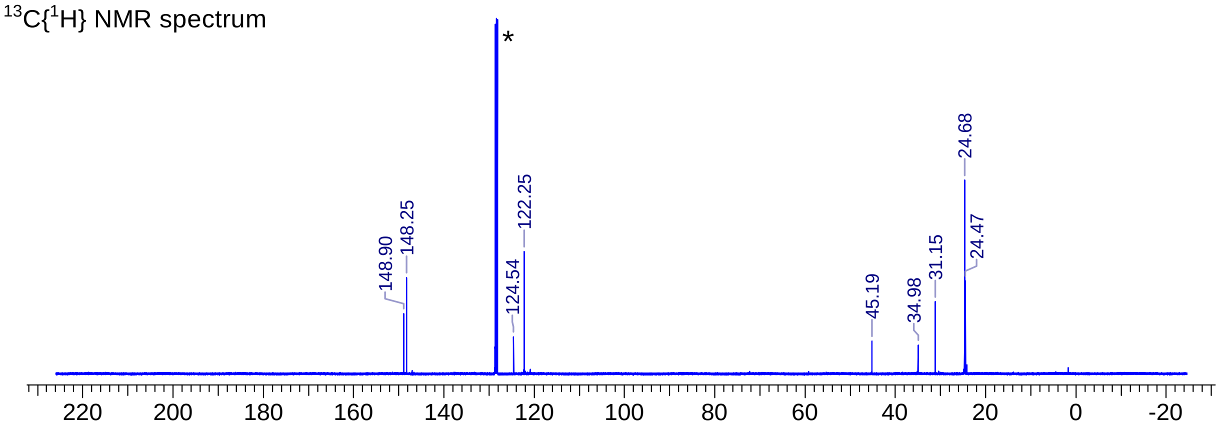

2.2 Synthesis of triazaarsoles

2.2.1 3-Benzyl-5-(trimethylsilyl)-3*H*-1,2,3,4-triazaarsole (2a)

A solution of dichloro((trimethylsilyl)methyl)arsane (**1b**, 1.42 g, 6.1 mmol, 1.0 eq), benzyl azide (0.89 g, 6.7 mmol, 1.1 eq) and DABCO (1.5 g, 13.4 mmol, 2.2 eq) in DME (5 mL) are heated to 80 °C and stirred for three hours, whereupon all volatile components are removed *in vacuo* (1 x 10^{-3} mbar). The workup is performed under non-inert conditions. The reaction mixture is dissolved in DCM (5 mL) and silica (5 g) is added. The resulting sluggish mixture is dried *in vacuo* and the solids are added to the top of a silica column (7 x 1 cm). Firstly, side products and unreacted benzyl azide are eluted with *n*-pentane (300 mL), this fraction is discarded. Subsequently, the product is eluted with *n*-pentane/EtOAc (1:1, 120 mL) and the solvents are removed *in vacuo*, yielding a yellowish oil. To solidify this oil, *n*-pentane (3 x 1 mL) is added and removed after the addition *in vacuo* (1 x 10^{-3} mbar). Product **2a** results as an off-white solid, which is slightly air- and moisture sensitive (yield: 0.44 g, 1.5 mmol, 24.6%). Crystals in form of colorless needles suitable for Xray crystallography were grown by storing saturated *n*-pentane solution of **2a** for three days at −18 °C.

M($C_{11}H_{16}AsN_3Si$) = 293.28 g/mol. **^{1}H NMR** (DCM-d_2, 399.74 MHz, 292.6 K) δ [ppm] = 7.40–7.30 (m, 5H, ArC*H*); 5.88 (s, 2H, BzCH_2); 0.37 (s, 9H, TMS-CH_3). **^{13}C{^{1}H} NMR** (DCM-d_2, 100.71 MHz, 293.5 K) δ [ppm] = 205.2 (s, ArAs*C*); 138.8 (s, Ar*C*); 129.5 (s, Ar*C*); 129.0 (s, Ar*C*); 128.9 (s, Ar*C*); 58.8 (s, Bz*C*H$_2$); 0.1 (br.s, TMS-*C*H$_3$). **^{29}Si{^{1}H}**

NMR (DCM-d_2, 79.57 MHz, 293.8 K) δ [ppm] = −5.25. **ESI-TOF (m/z):** 294.04 g/mol ($C_{11}H_{17}AsN_3Si^+$ = [M+H$^+$]).

Figure S5: NMR spectra of **2a** (DCM-d_2 solvent signals (^{1}H NMR: 5.32 ppm ^{13}C NMR: 54.0 ppm) and grease signals (^{1}H NMR: 0.08 ppm ^{13}C{^{1}H} NMR: 1.3 ppm ^{29}Si{^{1}H} NMR: −21.8 ppm) indicated by asterisks).

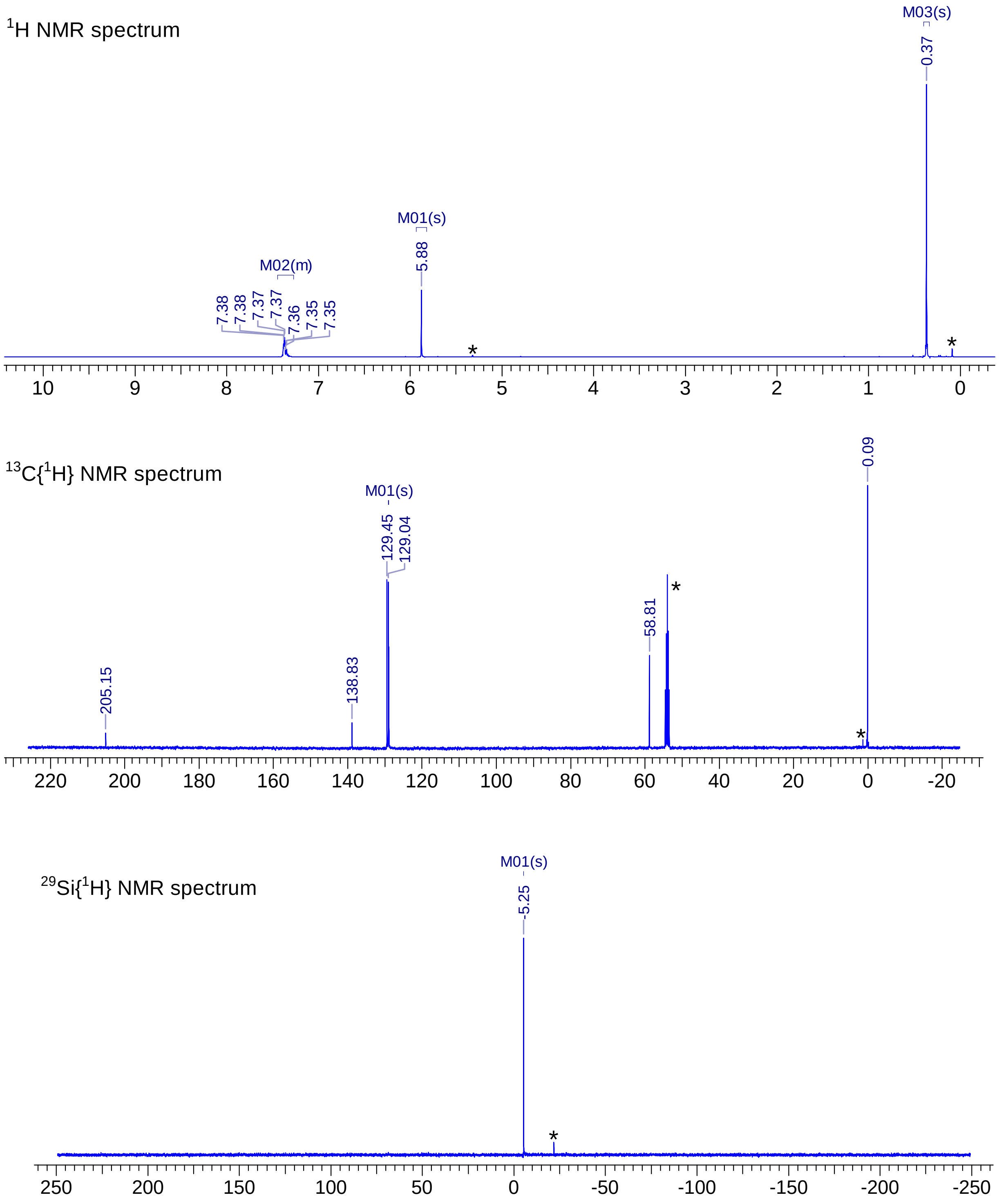

2.2.3 3-Benzyl-5-phenyl-3*H*-1,2,3,4-triazaarsole (2b)

Dichloro(phenyl(trimethylsilyl)methyl)arsane (**1a**, 0.20 g, 0.65 mmol, 1.0 eq) is dropped into a solution of DABCO (73 mg, 0.65 mmol, 1.0 eq) and benzyl azide (86 mg, 0.65 mmol, 1.0 eq) in Et_2O (5 mL) at room temperature and the resulting reaction mixture is stirred for 3 days. Afterwards, more Et_2O (3 mL) is added and the resulting suspension is filtered (*via* cannula filter) yielding a slightly reddish solution. All volatile components are removed *in vacuo* (1 x 10^{-3} mbar) and the resulting solids are washed with *n*-pentane (2 x 4 mL) to remove unreacted azide. Afterwards, the solids are dried *in vacuo* (1 x 10^{-3} mbar) and redissolved in Et_2O (10 mL). Subsequently, the organic fraction is washed with water (3 x 10 mL, the aqueous fraction was discarded) and dried over $MgSO_4$ (overnight for approx. 16 hours). Afterwards, the solvent is removed *in vacuo* (1 x 10^{-3} mbar) and the product **2b** resulted in form of a colourless solid which decomposes on air over time. Crystals of **2b** suitable for Xray crystallography can be grown from a saturated *n*-pentane solution *via* slow evaporation of the solvent at ambient pressure at room temperature. YIELD: (Yield: 170 mg, 0.57 mmol, 88%).

M($C_{14}H_{12}AsN_3$) = 297.20 g/mol. **^{1}H NMR** (DCM-d_2, 400.53 MHz, 292.8 K) δ [ppm] = 7.94–7.87 (m, 2H, ArC*H*); 7.45 – 7.33 (m, 8H, ArC*H*); 5.82 (s, 2H, BzCH_2). **^{13}C{^{1}H}-NMR** (DCM-d_2, 100.71 MHz, 293.9 K) δ [ppm] = 200.3 (s, ArAs*C*); 129.9 (s, Ar*C*); 129.6 (s, Ar*C*); 129.5 (s, Ar*C*); 129.0 (s, Ar*C*); 127.5 (s, Ar*C*); 59.4 (Bz*C*H_2). **ESI-TOF (m/z):** 297.20 g/mol ($C_{14}H_{12}AsN_3$ = [M]).

Figure S6: NMR and IR spectra of **2b** (DCM-d_2 solvent signals (^{1}H NMR: 5.32 ppm ^{13}C NMR: 54.0 ppm) and grease signals (^{1}H NMR: 0.08 ppm ^{13}C NMR: 1.3 ppm) indicated by asterisks).

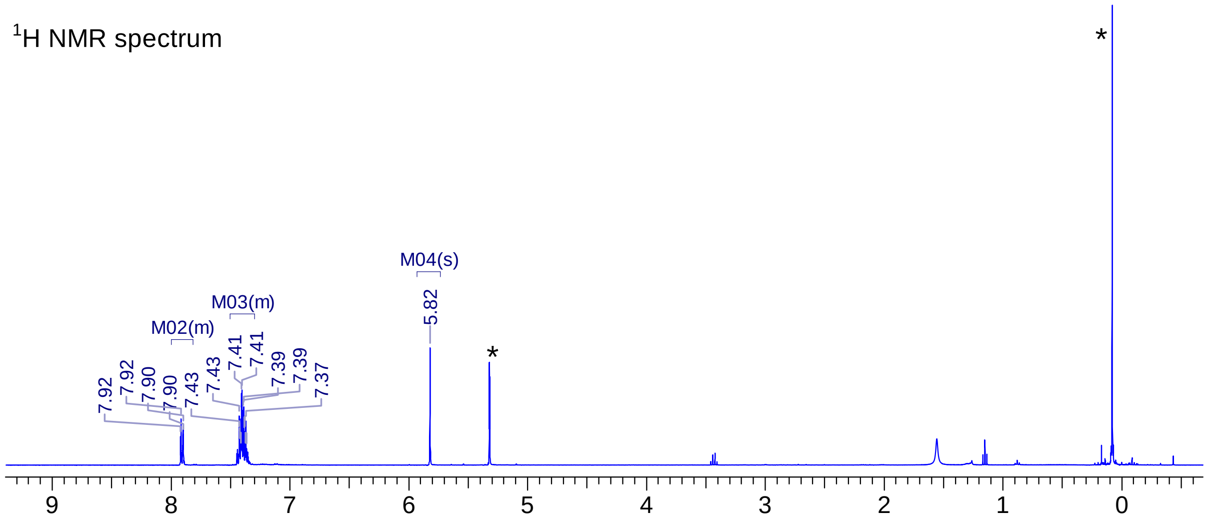

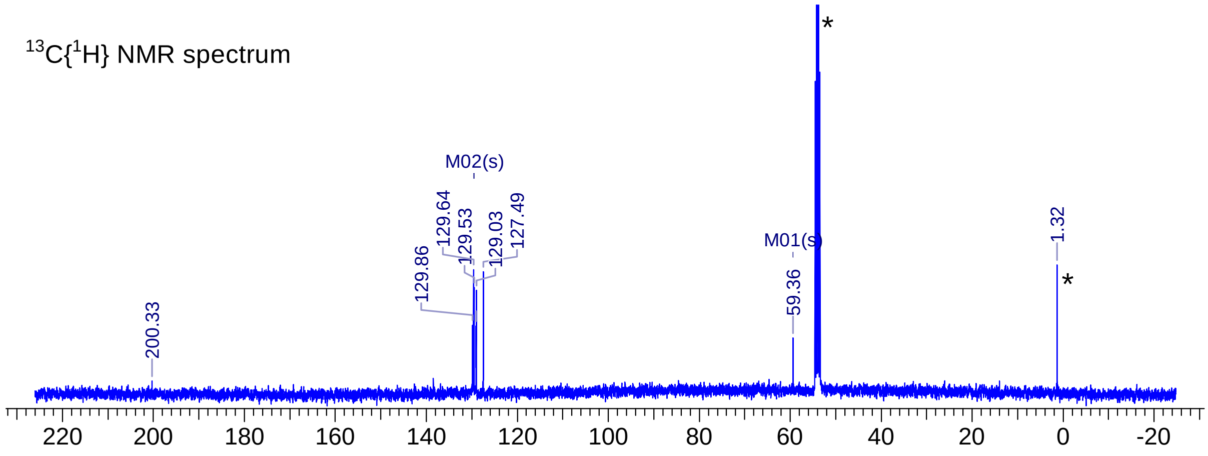

2.2.4 3-Benzyl-5-mesityl-3*H*-1,2,3,4-triazaarsole (2c)

Dichloro(2,4,6-trimethylbenzyl)arsane (**1c**, 2.00 g, 7.17 mmol, 1.0 eq) is added to a solution of DABCO (1.77 g, 15.8 mmol, 2.2 eq) and benzyl azide (0.95 g, 7.17 mmol, 1.0 eq) in DME (40 mL), whereupon the mixture turns slightly yellow, and the formation of a white precipitate can be observed. The mixture is stirred at 85 °C for 50 hours and subsequently, all volatile components are removed *in vacuo*, yielding yellowish solids. The workup is performed under non-inert conditions. Et_2O (50 mL) is added to the solids and the resulting suspension is filtered using a celite-packed frit. The solids are washed with Et_2O (2 x 30 mL) and the filtrate is reduced *in vacuo* (1 x 10^{-3} mbar, to a volume of approx. 5 mL). To this residue, silica (approx. 5 g) is added and the resulting sluggish mixture is dried *in vacuo* (1 x 10^{-3} mbar). The resulting solids are added to the top of a silica column (13 x 3 cm). Firstly, side products and unreacted benzyl azide are eluted with *n*-pentane (1000 mL), this fraction is discarded. Subsequently, the product is eluted with DCM (1500 mL) and the solvents are removed *in vacuo* (1 x 10^{-3} mbar), yielding the product **2c** as off-white slightly air and moisture sensitive solid (yield: 1.1 g, 3.2 mmol, 45%). Crystals suitable for Xray crystallography can be grown by slowly cooling down a saturated *n*-pentane solution to −30 °C, yielding colorless plates of **2c**.

M($C_{17}H_{18}AsN_3$) = 339.26 g/mol. **^{1}H NMR** ($CDCl_3$, 399.74 MHz, 294 K) δ [ppm] = 7.48–7.38 (m, 5H, ArC*H*); 6.97 (s, 2H, ArC*H*); 5.88 (s, 2H, BzCH_2); 2.33 (s, 3H, MesCH_3); 2.11 (s, 6H, MesCH_3). **^{13}C{^{1}H} NMR** ($CDCl_3$, 101 MHz, 295.9 K) δ [ppm] = 198.4 (s, ArAs*C*); 138.1 (s, Ar*C*); 137.6 (s, Ar*C*); 136.1 (s, Ar*C*); 132.0 (s, Ar*C*); 129.1 (s, Ar*C*);

128.6 (s, Ar*C*); 59.0 (s, Bz*C*H_2); 21.5 (Mes*C*H_3); 21.2(Mes*C*H_3). **ESI-TOF (m/z):** 340.08 g/mol ($C_{17}H_{18}AsN_3^+$ = [M+H$^+$]).

Figure S7: NMR spectra of **2c** ($CDCl_3$ solvent signals (^{1}H NMR: 7.27 ppm ^{13}C NMR: 77.0 ppm) indicated by asterisks).

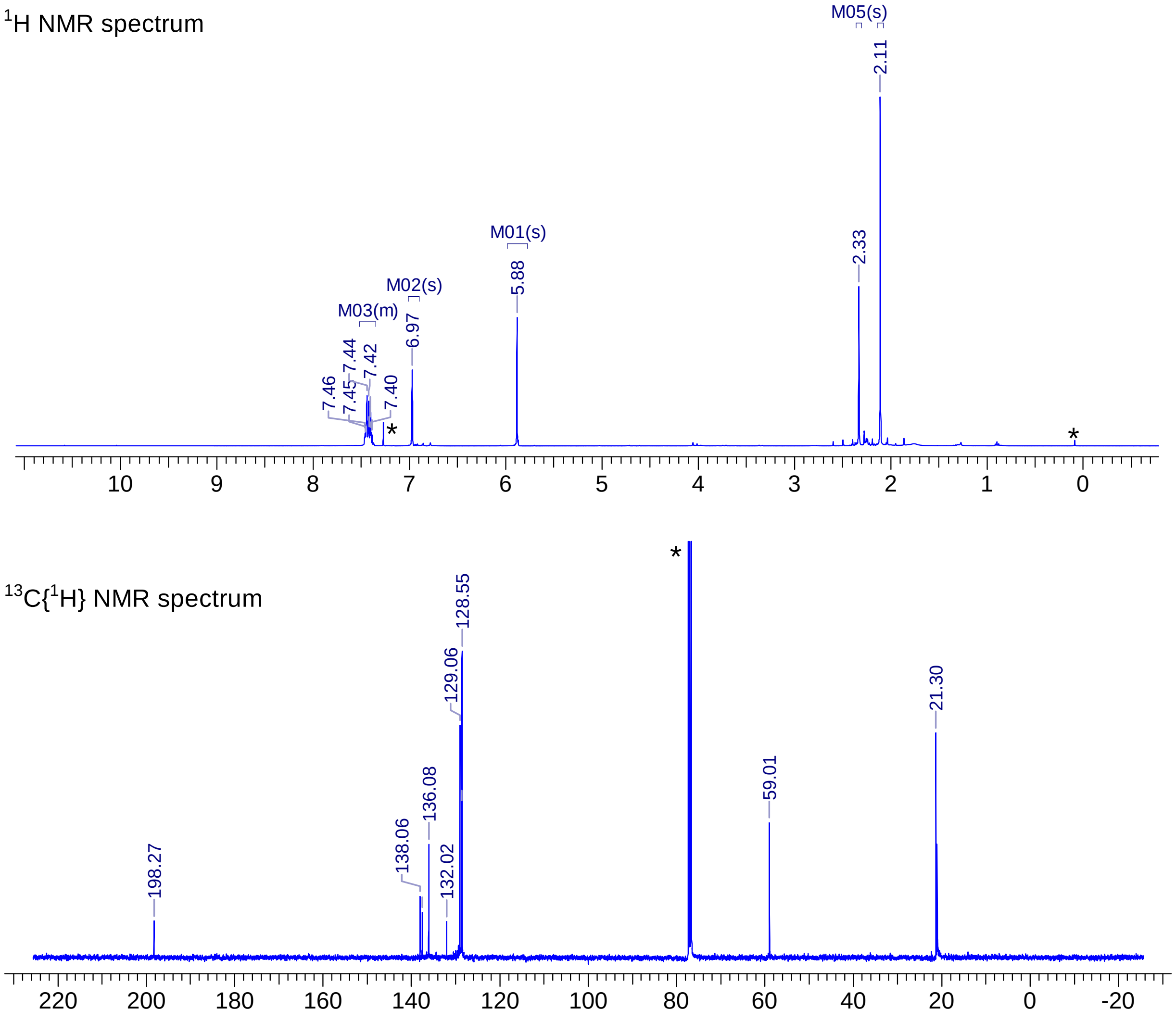

2.2.5 3-Benzyl-5-(2,4,6-tri*iso*propylphenyl)-3*H*-1,2,3,4-triazaarsole (2d)

DME (10 mL) is added to a solution of dichloro(2,4,6-tri*iso*propylbenzyl)arsane (**1d**, 0.80 g, 2.2 mmol, 1.0 eq), DABCO (0.54 g, 4.8 mmol, 2.2 eq) and KI (0.73 g, 4.4 mmol, 2.0 eq). Subsequently, benzyl azide (0.30 g, 2.2 mmol, 1.0 eq) is added at room temperature. The resulting turbid mixture is stirred for 48 hours at 80 °C, whereupon the mixture turns brown, and a brownish precipitate is formed. The workup is performed under non-inert conditions. Afterwards, all volatile components are removed *in vacuo* (1 x 10^{-3} mbar). The resulting residues are added to the top of a silica column (10 x 1 cm). Firstly, side products and unreacted benzyl azide are eluted with *n*-hexane (200 mL), this fraction is discarded. Subsequently, the product is eluted with DCM

(150 mL) and the solvent is removed *in vacuo* (1 x 10^{-3} mbar), yielding **2d** as an off-white solid (yield 370 mg, 0.90 mmol, 41%).

M($C_{23}H_{30}AsN_3$) = 423.16 g/mol. **^{1}H NMR** (C_6D_6, 399.37 MHz, 291.8 K) δ [ppm] = 7.22 (s, 2H, ArC*H*); 7.00–7.10 (m, 2H, ArC*H*); 6.92–7.03 (m, 3H, ArC*H*); 5.36 (s, 2H, BzC*H*$_2$); 2.98 (hept, *J* = 6.9 Hz, 1H, *i*PrC*H*); 2.85 (hept, *J* = 6.8 Hz, 2H, *i*PrC*H*); 1.28 (d, *J* = 6.9 Hz, 6H, Tripp-C*H*$_3$); 1.16 (d, *J* = 6.9 Hz, 12H, Tripp-C*H*$_3$). **^{13}C{^{1}H} NMR** (C_6D_6, 100.42 MHz, 292.8 K) δ [ppm] = 199.5 (s, ArAs*C*); 150.1 (s, Ar*C*); 147.8 (s, Ar*C*); 138.4 (s, Ar*C*); 130.9 (s, Ar*C*); 129.5 (s, Ar*C*); 129.1 (s, Ar*C*); 128.9 (s, Ar*C*); 121.4 (s, Ar*C*); 59.2 (s, Bz*C*H$_2$); 35.3 (s, *i*Pr*C*H); 31.2 (s, *i*Pr*C*H$_3$); 24.8 (s, *i*Pr*C*H$_3$); 24.7 (s, *i*Pr*C*H$_3$). **ESI-TOF (m/z):** 423.15 g/mol ($C_{23}H_{30}AsN_3$ = [M]).

Figure S8: NMR spectra of **2d** (DCM-*d*$_2$ solvent signals (^{1}H NMR: 5.32 ppm ^{13}C NMR: 54.0 ppm) and grease signals (^{1}H NMR: 0.08 ppm ^{13}C{^{1}H} NMR: 1.3 ppm) indicated by asterisks).

^{1}H NMR spectrum

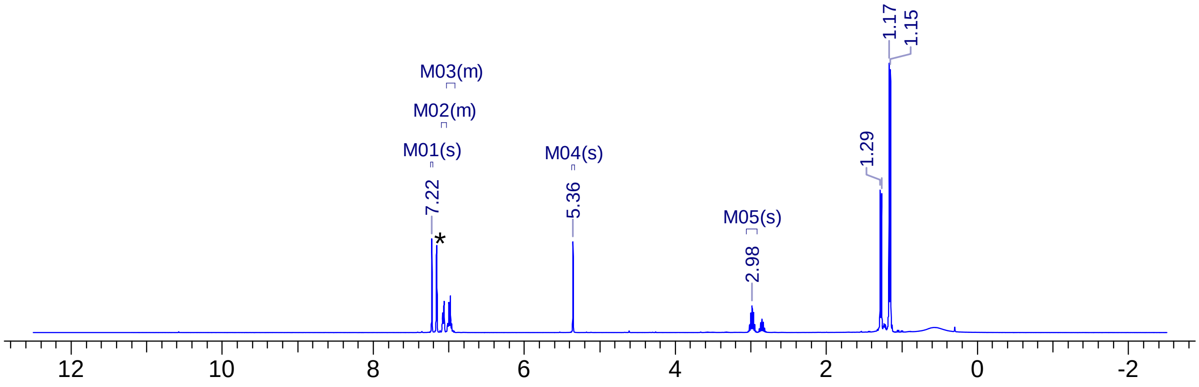

^{13}C{^{1}H} NMR spectrum

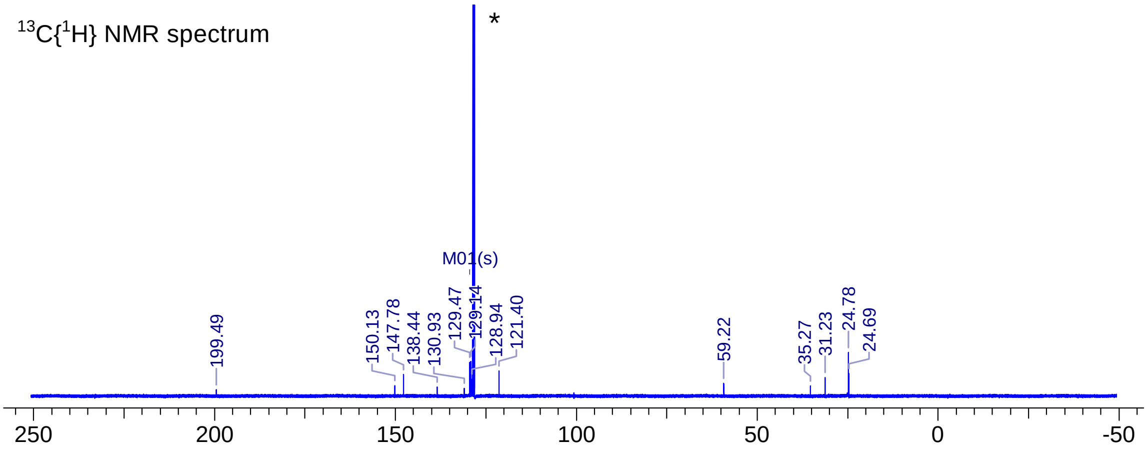

2.2.6 3-Mesityl-5-(trimethylsilyl)-3*H*-1,2,3,4-triazaarsole (2e)

Dichloro((trimethylsilyl)methyl)arsane (**1b**, 2.3 g, 9.9 mmol, 1.0 eq) is added to a solution of DABCO (2.4 g, 21.8 mmol, 2.2 eq) and mesityl azide (1.6 g, 9.9 mmol, 1.0 eq) in DME (60 mL) at room temperature, whereupon the formation of a colorless precipitate is observed. The mixture is stirred for one day at 85 °C. Afterwards, all volatile components are removed *in vacuo* (1 x 10^{-3} mbar). The resulting solids are dissolved in DCM (2 mL) and added to the top of an inert silica column (12 x 3 cm). The product is eluted with DCM (250 mL) and the solvent is removed *in vacuo* (1 x 10^{-3} mbar), yielding a brownish, viscous residue. The residue is dissolved in *n*-pentane and the solution is stored at −18 °C, yielding the product **2e** in form of an air- and moisture sensitive slightly off-white crystalline solid (yield: 2.1 g, 6.5 mmol, 66%). Crystals suitable for Xray crystallography can be grown by slowly cooling down a saturated *n*-pentane solution to −30 °C.

M($C_{13}H_{20}AsN_3Si$) = 321.33 g/mol. **^{1}H NMR** (DCM-*d*$_2$, 400.53 MHz, 294.3 K) δ [ppm] = 7.03 (s, 2H, Mes-ArC*H*); 2.37 (s, 3H, MesC*H*$_3$); 1.90 (s, 6H, Mes-C*H*$_3$); 0.47 (s, 9H, TMS-C*H*$_3$). **^{13}C{^{1}H} NMR** (DCM-*d*$_2$, 100.71 MHz, 294.1 K) δ [ppm] = 206.5 (s, ArAs*C*); 139.6 (s, Ar*C*); 139.3 (s, Ar*C*); 134.7 (s, Ar*C*); 129.6 (s, Ar*C*); 21.4 (s, Mes*C*H$_3$); 18.1 (s, Mes*C*H$_3$); 0.2 (s, TMS-*C*H$_3$). **^{29}Si{^{1}H} NMR** (DCM-*d*$_2$, 79.57 MHz, 294.1 K) δ [ppm] = −5.1 (TMS). **ESI-TOF (m/z):** 322.0745 g/mol ($C_{13}H_{21}AsN_3Si^+$ = [M+H$^+$]).

Figure S9: NMR spectra of **2e** (DCM-*d*$_2$ solvent signals (^{1}H NMR: 5.32 ppm ^{13}C NMR: 54.0 ppm) and grease signals (^{1}H NMR: 0.08 ppm ^{13}C{^{1}H} NMR: 1.3 ppm ^{29}Si{^{1}H} NMR: −21.8 ppm) indicated by asterisks).

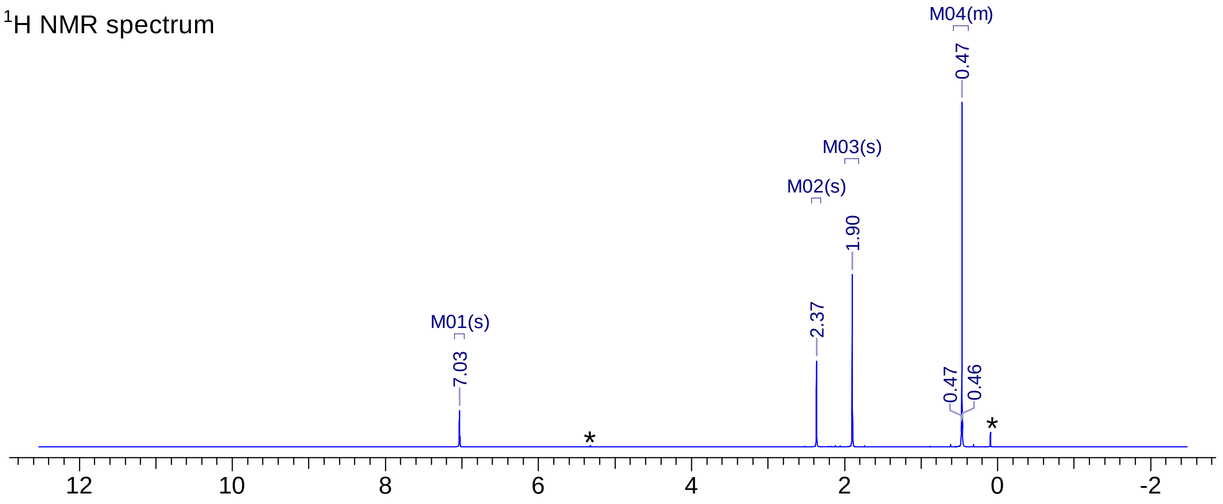

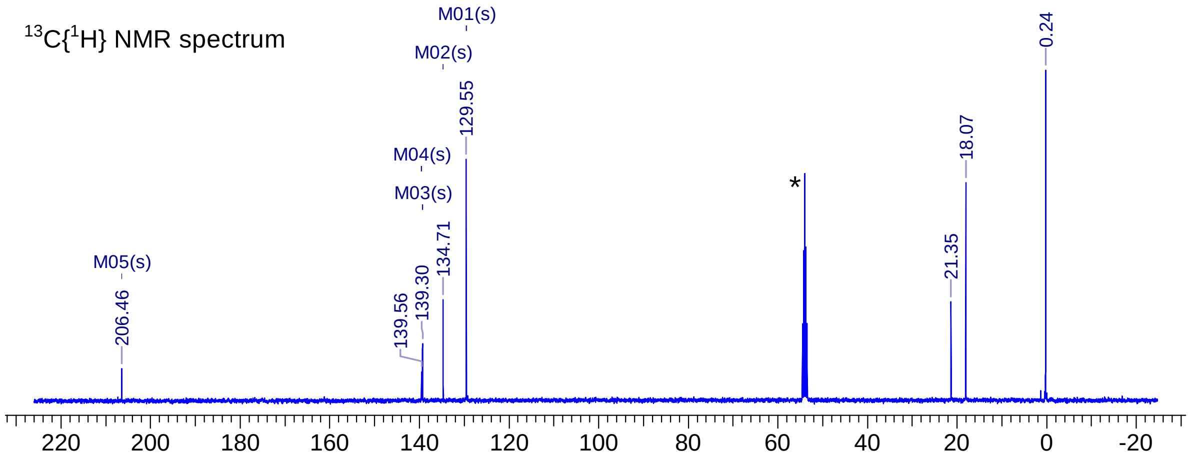

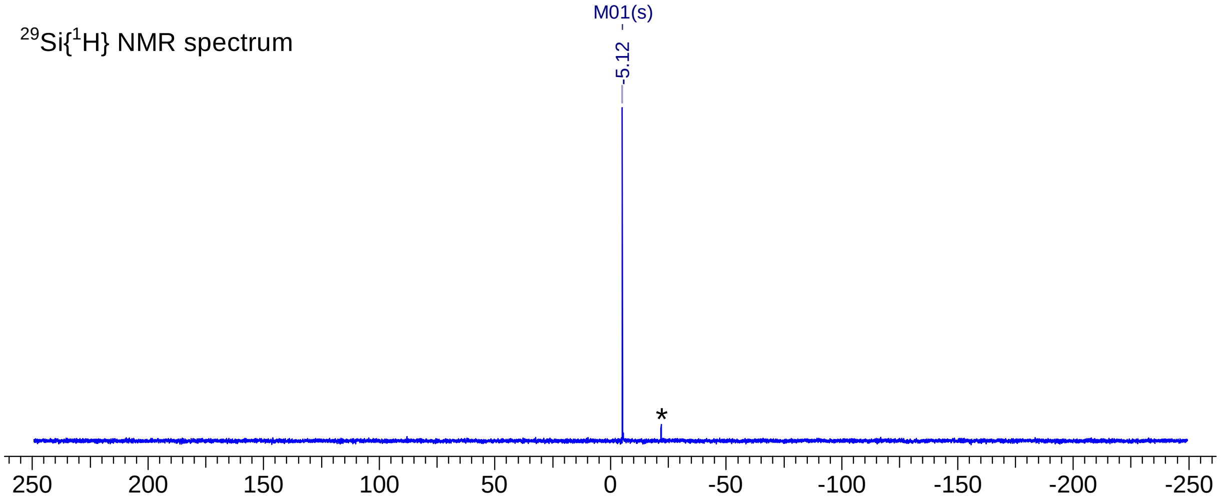

2.2.7 3,5-Dimesityl-3*H*-1,2,3,4-triazaarsole (2f)

Dichloro(2,4,6-trimethylbenzyl)arsane (**1c**, 1.99 g, 7.15 mmol, 1.0 eq) is added to a solution of DABCO (1.77 g, 15.8 mmol, 2.2 eq) and mesityl azide (1.15 g, 7.15 mmol, 1.0 eq) in DME (40 mL) at room temperature, whereupon a colorless precipitate is formed. The mixture is stirred for 50 hours at 85 °C, and subsequently, all volatile components are removed *in vacuo* (1 x 10^{-3} mbar), yielding off-white solids. The workup is performed under non-inert conditions. Et_2O is added to the solids and filtered (P4 glas frit). The solids are washed with Et_2O (2 x 30 mL) and the filtrate is reduced *in vacuo* (1 x 10^{-3} mbar, to approx. 5 ml). To this residue, silica (approx. 5 g) is added and the resulting sluggish mixture is dried *in vacuo* (1 x 10^{-3} mbar). The resulting solids are added to the top of a silica column (7 x 1 cm). Firstly, side products and unreacted mesityl azide are eluted with *n*-pentane (350 mL), this fraction is discarded. Subsequently, the product is eluted with DCM (300 mL) and the solvents are removed *in vacuo*, yielding the product **2f** as off-white slightly air and moisture sensitive solid

(yield: 1.68 g, 4.6 mmol, 64%). Crystals suitable for Xray crystallography can be grown by slowly cooling down a saturated solution of **3f** in acetonitrile to −30 °C.

M($C_{19}H_{22}AsN_3$) = 367.31 g/mol. **^{1}H NMR** ($CDCl_3$, 400.53 MHz, 294.4 K) δ [ppm] = 7.05 (s, 2H, ArC*H*); 7.02 (s, 2H, ArC*H*); 2.39 (s, 3H, MesC*H*$_3$); 2.36 (s, 3H, MesC*H*$_3$); 2.20 (s, 6H, MesC*H*$_3$); 2.05 (s, 6H, MesC*H*$_3$). **^{13}C{^{1}H} NMR** ($CDCl_3$, 100.71 MHz, 294.2 K) δ [ppm] = 199.6 (s, ArAs*C*); 138.9 (s, Ar*C*); 138.7 (s, Ar*C*); 138.2 (s, Ar*C*); 136.0 (s, Ar*C*); 134.2 (s, Ar*C*); 131.8 (s, Ar*C*); 129.1 (s, Ar*C*); 128.6 (s, Ar*C*); 21.2 (s, Mes*C*H$_3$); 21.1 (s, Mes*C*H$_3$); 21.0 (s, Mes*C*H$_3$); 17.6 (Mes*C*H$_3$). **ESI-TOF (m/z):** 368.1128 g/mol ($C_{19}H_{23}AsN_3^+$ = [M+H$^+$]).

Figure S10: NMR spectra of **2f** ($CDCl_3$ solvent signals (^{1}H NMR: 7.27 ppm ^{13}C NMR: 77.0 ppm) indicated by asterisks).

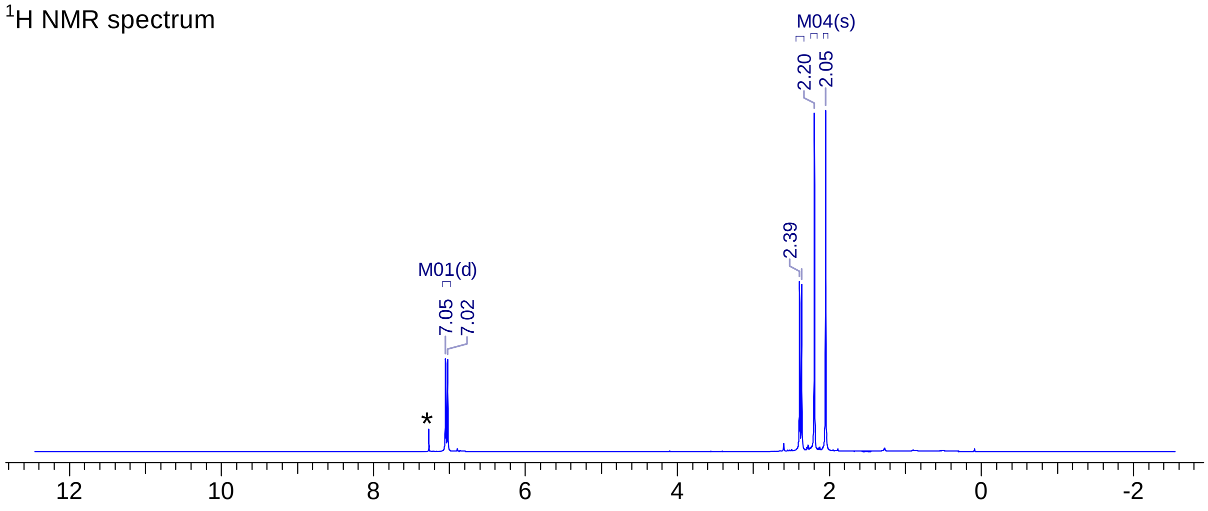

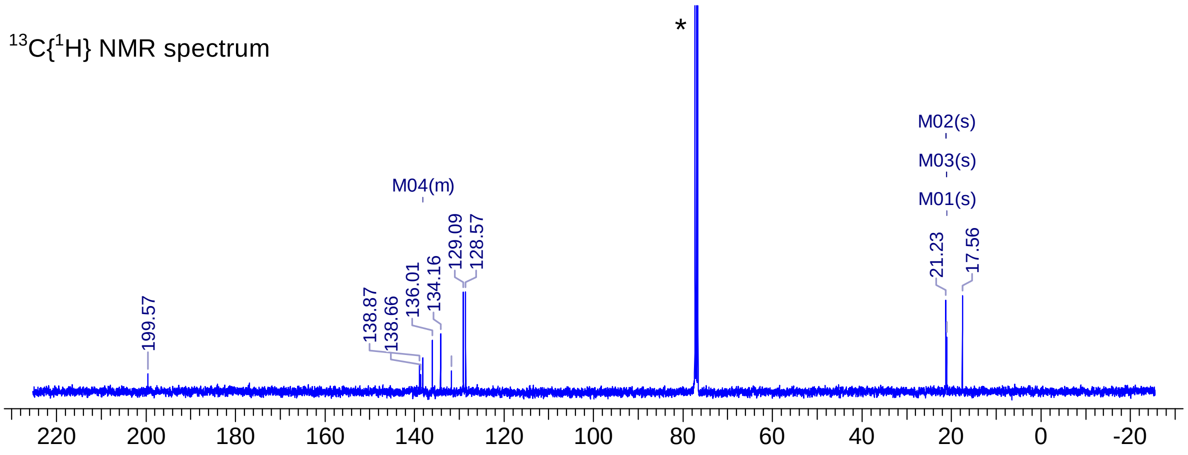

2.2.8 3-(2,6-di*iso*propylphenyl)-5-phenyl-3*H*-1,2,3,4-triazaarsole (2g)

KI (700.6 mg, 4.22 mmol, 2 eq) is dissolved in DME (17 mL) and dichloro(phenyl(trimethylsilyl)methyl)arsane (**1a**, 0.653 g, 2.1 mmol, 1 eq) and 2-azido-1,3-di*iso*propylbenzene (0.427 g, 2.1 mmol, 1.0 eq) are added. The reaction mixture is heated to 85 °C and DABCO (0.493 g, 4.4 mmol, 2.1 eq) is added. The reaction mixture is stirred at 85 °C for six days. Subsequently all volatile components are removed *in vacuo* (1 x 10^{-3} mbar) and the residue is purified *via* column chromatography (non-inert). Thereby, side products and unreacted azide is eluted first (500 ml *n*-pentane, this fraction is discarded) and afterwards the product is extracted using DCM (250 mL). All volatile components of the DCM fraction are removed *in vacuo* (1 x 10^{-3} mbar) and the raw product is washed with cold *n*-pentane (2 x 3 mL, 0 °C). The product is isolated in form of a white crystalline precipitate (yield: 0.355 g, 0.967 mmol, 45.8%) Crystals of **2g** suitable for X-Ray analysis can be grown from a saturated *n*-pentane/DCM solution (1:1 mixture) at room temperature.

M($C_{19}H_{22}AsN_3$) = 367.10 g/mol. **^{1}H NMR** (DCM-d_2, 400.53 MHz, 293.7 K) δ [ppm] = 8.07 (br. d, J = 7.3 Hz, 2H, ArC*H*); 7.55–7.40 (m, 5H, ArC*H*); 7.35–7.30 (m, 3H, ArC*H*); 2.45–2.30 (m, 2H, *i*PrC*H*); 2.45–2.30 (m, 12H *i*PrC*H*$_3$). **^{13}C{^{1}H} NMR** (DCM-d_2, 100.71 MHz, 294.2 K) δ [ppm] = 201.1 (s, ArAs*C*); 145.4 (s, Ar*C*); 137.8 (s, Ar*C*); 136.2 (s, Ar*C*); 129.9 (s, Ar*C*); 129.6 (s, Ar*C*); 129.3 (s, Ar*C*); 127.1 (s, Ar*C*); 123.7 (s, Ar*C*); 28.2 (s, *i*Pr*C*H); 24.1 (s, *i*Pr*C*H$_3$); 24.0 (s, *i*Pr*C*H$_3$). **ESI-TOF (m/z):** 368.11 g/mol ($C_{19}H_{23}AsN_3^+$ = [M+H$^+$]).

Figure S11: NMR spectra of **2g** (DCM-d_2 solvent signals (^{1}H NMR: 5.32 ppm ^{13}C NMR: 54.0 ppm) indicated by asterisks).

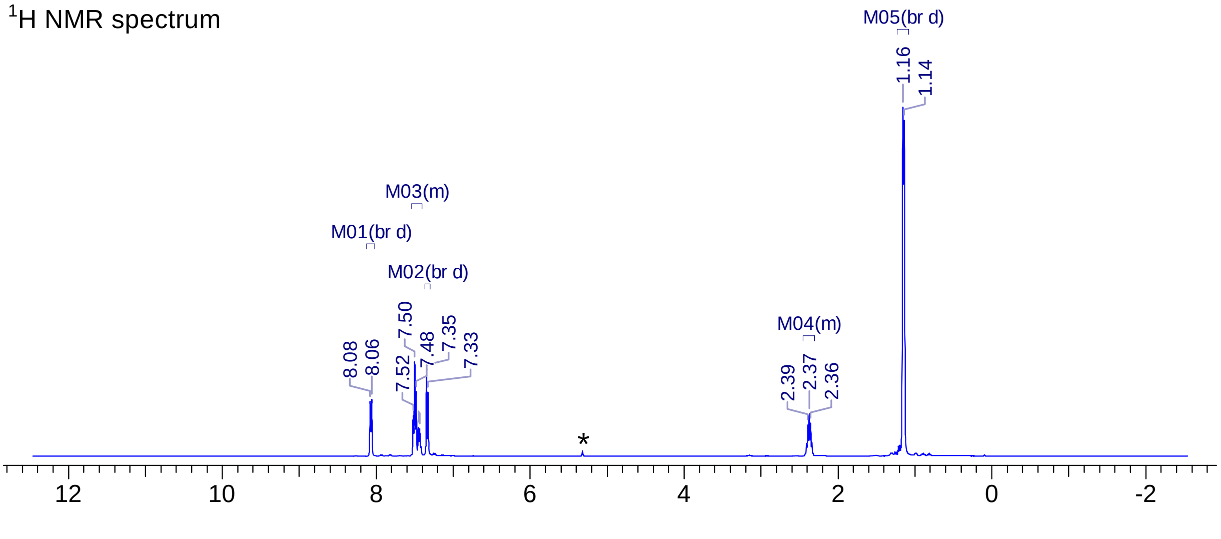

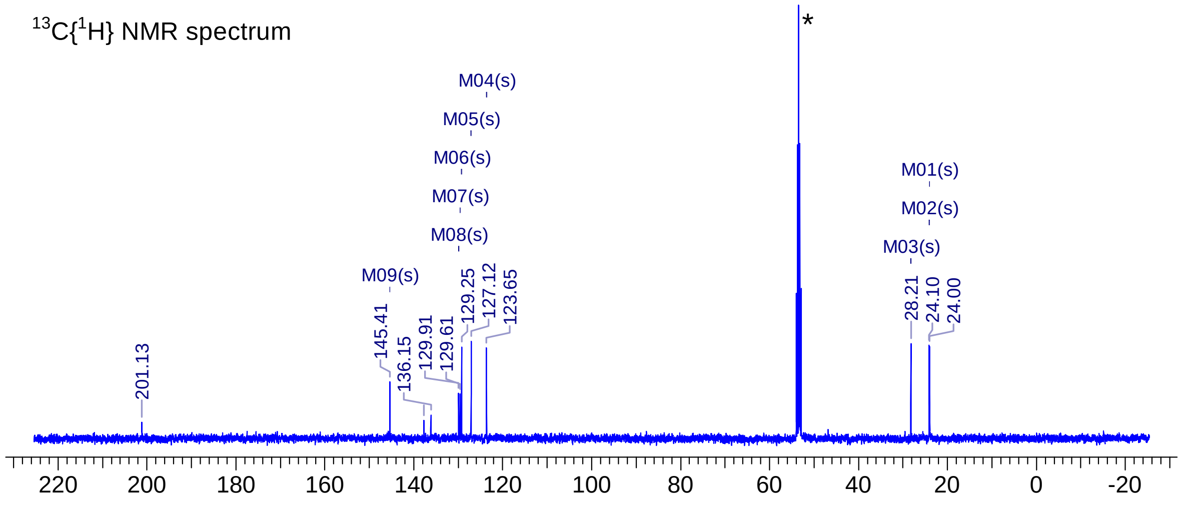

2.2.9 3-(2,6-Di*iso*propylphenyl)-5-(2,4,6-tri*iso*propylphenyl)-3*H*-1,2,3,4-triaza-arsole (2h)

Dichloro(2,4,6-tri*iso*propylbenzyl)arsane (**1d**, 2.0 g, 5.5 mmol, 1.0 eq.) are combined with KI (1.83 g, 11 mmol, 2.0 eq.), DABCO (1.30 g, 11.6 mmol, 2.1 eq.) and 2-azido-1,3-di*iso*propylbenzene (1.12 g, 5.5 mmol, 1.0 eq.). DME (40 ml) is added, and the resulting suspension is stirred for two days at 85 °C. Afterwards, all volatile components are removed *in vacuo* (1 x 10^{-3} mbar) and the raw product is purified *via* non-inert column chromatography using silica (3 x 20 cm). The unreacted azide is removed by washing with *n*-hexane in a first step (this fraction is discarded), and in a second step the product is eluted using DCM. All volatile components of the DCM fraction are removed *in vacuo* (1 x 10^{-3} mbar) and the product results in form of a slightly yellowish powder, which is again washed with cold *n*-pentane (−100 °C, 30 ml). The product **2h** results in form of a colorless powder (1.14 g; 2.31 mmol; 42%)

Mw($C_{28}H_{40}AsN_3$) = 493.56 g/mol. **^{1}H NMR** (C_6D_6, 600.17 MHz, 293.2 K) δ [ppm] = 7.28 (s, 2H, ArC*H*); 7.22–7.67 (m, 1H, ArC*H*); 7.10–7.14 (m, 2H, ArC*H*); 3.10–3.20 (m, 2H, *i*PrC*H*); 2.85–2.95 (m, 1H, *i*PrC*H*); 2.60–2.68 (m, 2H, *i*PrC*H*); 1.31 (d, *J* = 6.9 Hz, 6H, *i*PrCH_3); 1.25 (d, *J* = 6.9 Hz, 12H, *i*PrCH_3); 1.12 (d, *J* = 6.9 Hz, 6H, *i*PrCH_3); 1.08 (d, *J* = 6.9 Hz, 6H, *i*PrCH_3). **^{13}C{^{1}H} NMR** (C_6D_6, 150.91 MHz, 293.2 K) δ [ppm] = 201.0 (s, ArAs*C*); 150.4 (s, Ar*C*); 147.6 (s, Ar*C*); 145.9 (s, Ar*C*); 139.1 (s, Ar*C*); 129.6 (s, Ar*C*); 129.5 (s, Ar*C*); 128.7 (s, Ar*C*); 123.6 (s, Ar*C*); 120.8 (s, Ar*C*); 35.3 (s, *i*Pr*C*H); 31.5 (s, *i*Pr*C*H); 29.2 (s, *i*Pr*C*H); 24.7 (br. s, *i*Pr*C*H$_3$). **ESI-TOF (m/z):** 494.29 g/mol ($C_{28}H_{41}AsN_3^+$ = [M+H$^+$]).

Figure S12: NMR spectra of **2h** (DCM-d_2 solvent signals (^{1}H NMR: 5.32 ppm ^{13}C NMR: 54.0 ppm) indicated by asterisks).

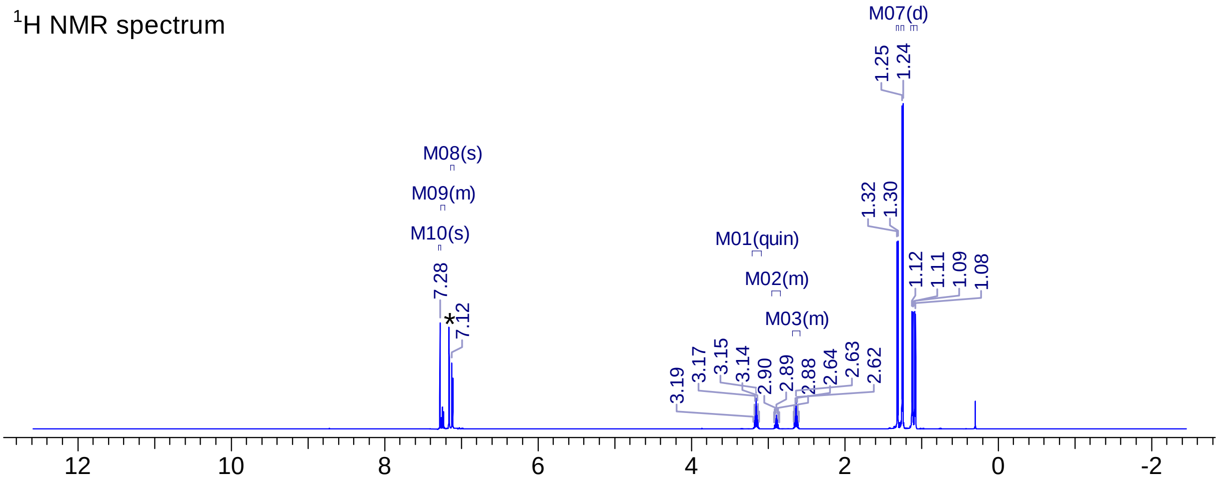

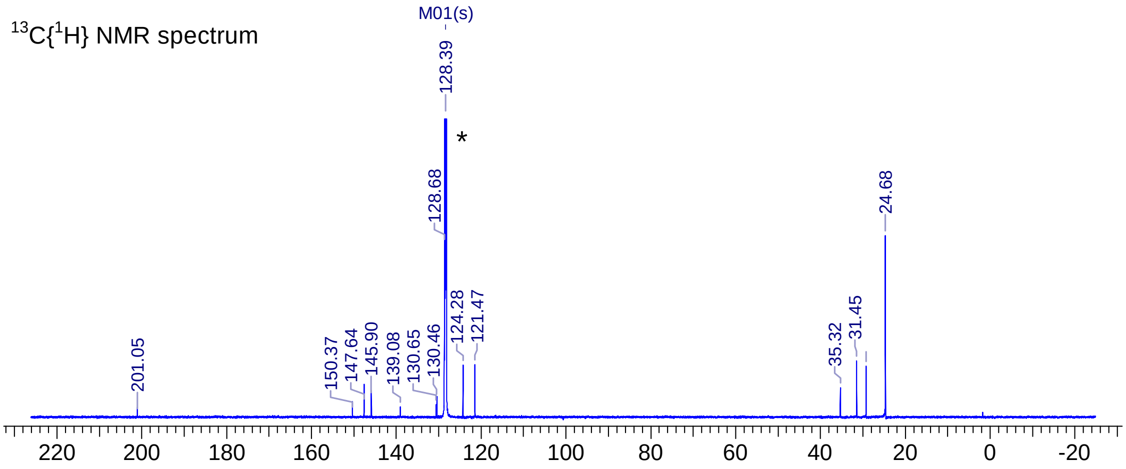

2.2.9 3-Mesityl-5-(terphenyl)-3*H*-1,2,3,4-triazaarsole (2i)

Dichloro(2,4,6-trimethylbenzyl)arsane (**1c**, 0.58 g, 2.08 mmol, 1.50 eq.) and terphenyl azide (2,6-dimesitylphenyl azide, 0.49 g, 1.38 mmol, 1.0 eq.) are combined with KI (0.46 g, 2.76 mmol, 2.0 eq.) and DABCO (0.34 g, 3.04 mmol, 2.20 eq.). DME (20 ml) is added, and the resulting suspension is stirred for two days at 80 °C. Afterwards, the resulting slurry suspension is filtered (cannula filter) and all volatile components are removed *in vacuo* (1 x 10^{-3} mbar). To the resulting solids, *n*-pentane is added (10 ml) and the resulting suspension is filtered (cannula filter). The resulting beige, clear solution is stored in the freezer overnight (−30 °C) to give **2i** in form of beige crystals (suitable for Xray crystallography). The crystalline product **2i** is isolated, dried *in vacuo* (1 x 10^{-3} mbar) and the supernatant is discarded (0.28 g, 0.50 mmol; 36.2%).

Mw($C_{34}H_{36}AsN_3$) = 561.60 g/mol. **^{1}H NMR** (C_6D_6, 399.37 MHz, 291.9 K) δ [ppm] = 7.18–7.25 (m, 1H, ArC*H*); 7.07–7.11 (m, 2H, ArC*H*); 6.73 (s, 2H, ArC*H*); 6.67 (s, 4H, ArC*H*); 2.18 (s, 12H, *i*PrC*H*); 2.10 (s, 3H, *i*PrC*H*); 2.08 (s, 6H, *i*PrC*H*); 1.81 (s, 6H, *i*PrC*H*). **^{13}C{^{1}H} NMR** (C_6D_6, 100.71 MHz, 296.4 K) δ [ppm] = 198.0 (s, ArAs*C*); 141.3 (s, Ar*C*); 139.7 (s, Ar*C*); 137.9 (s, Ar*C*); 137.5 (s, Ar*C*); 136.8 (s, Ar*C*); 136.5 (s, Ar*C*); 135.6 (s, Ar*C*); 133.1 (s, Ar*C*); 130.1 (s, Ar*C*); 129.8 (s, Ar*C*); 129.0 (s, Ar*C*); 128.5 (s, Ar*C*); 34.8 (s, *i*Pr*C*H), 23.1 (s, *i*Pr*C*H_3); 21.5 (s, *i*Pr*C*H_3); 21.3 (s, *i*Pr*C*H_3); 21.2 (s, *i*Pr*C*H_3). **ESI-TOF (m/z):** 462.22 g/mol ($C_{34}H_{37}AsN_3^{+}$ = [M+H^{+}]).

Figure S13: NMR and IR spectra of **2i** (C_6D_6 solvent signals (^{1}H NMR: 7.16 ppm, ^{13}C NMR: 128.4 ppm) and *n*-pentane signals (^{1}H NMR: 0.87 ppm, 1.27 ppm, ^{13}C NMR: 14.3 ppm, 22.7 ppm) indicated by asterisks).

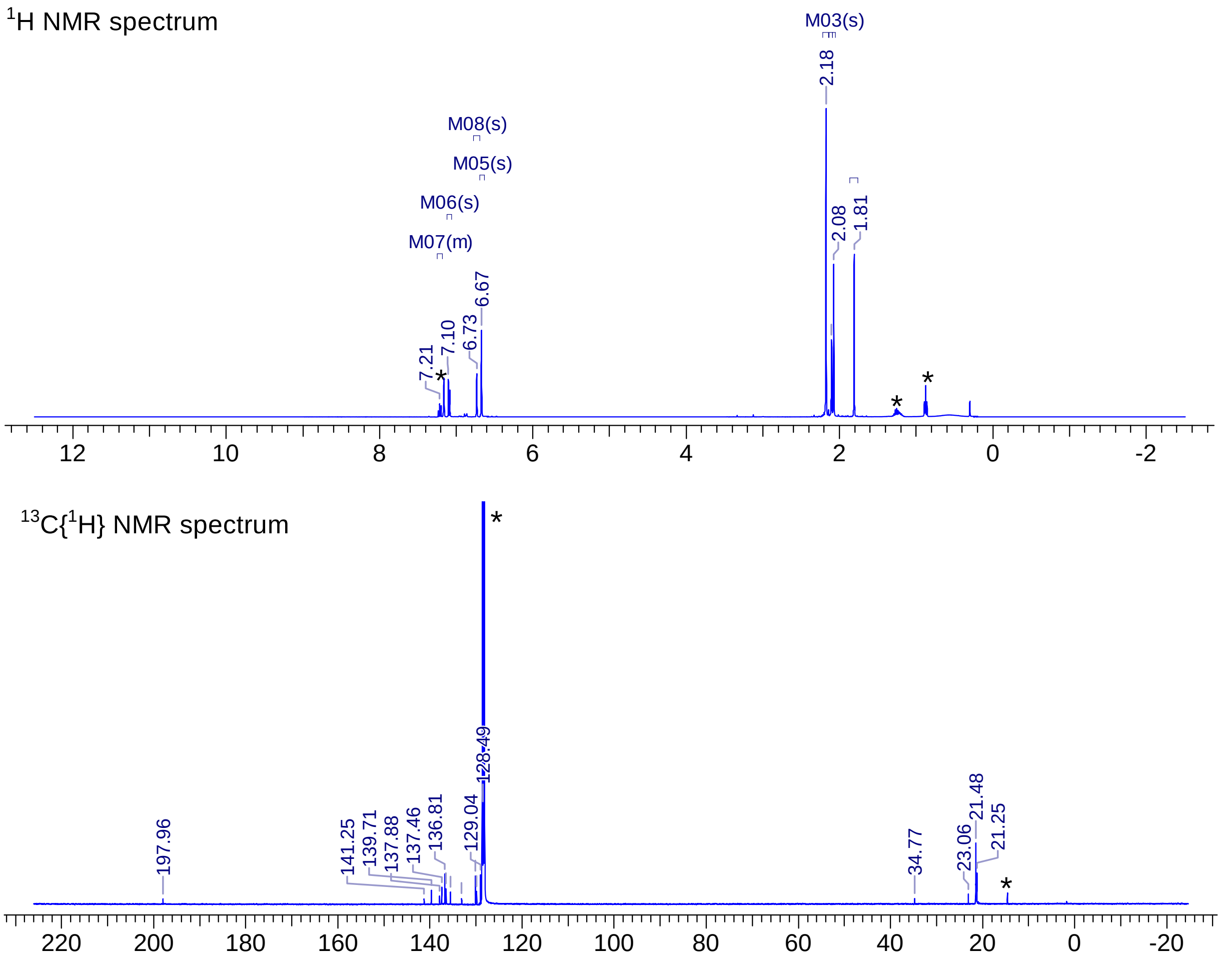

Please note: We also converted the following combinations of azides and chloroarsanes, under the above-mentioned reaction conditions: $MesN_3/PhCH_2AsCl_2$; $MesN_3/TrippCH_2AsCl_2$; $DippN_3/TMSCH_2AsCl_2$; $DippN_3/MesCH_2AsCl_2$. However, in these cases, some side products were formed, and we were not able to separate the products and the side-products to isolate pure product (presumably due to very comparable solubility properties). Thus, the synthesis procedures are not indicated in this manuscript. In case of converting $TerN_3/TrippCH_2AsCl_2$ we found that the formation of side products largely overcompensated the product formation, which we attribute in this case to the steric demand of the reactants involved. Even though single crystals of the desired compounds could be isolated and characterized, bulk phase material was not isolable, thus this synthetic procedure is also not indicated here.

3 Computational Details

Computations were carried out using Gaussian16.[61] Structure optimizations employed the pure DFT functional PBE[62,63] in conjunction with Grimme's dispersion correction D3(BJ)[64,65] and the def2-TVP basis set[66] (notation PBE-D3/def2-TVP). The resolution of identity (RI) approximation was applied using Weigend's accurate Coulomb-fitting basis set.[67] All structures were fully optimized and confirmed as minima by frequency analyses. Chemical shifts were derived by the GIAO method.[68-72] Please note that all computations were carried out for single, isolated molecules in the gas phase (ideal gas approximation). There may well be significant differences between gas phase and condensed phase.

3.1 Summary of calculated data

Table S2. Summary of calculated data, including electronic energies and thermal corrections (in a.u.).

Cmpd.	PG	N_{im}	$\langle S^2 \rangle$	E_{tot}	U_0	U_{298}	H_{298}	G_{298}
**N**N(H)N_2C(H)	C_S	0	0	−258.0901	−258.0442	−258.0407	−258.0397	−258.0703
**P**N(H)N_2C(H)	C_S	0	0	−544.6033	−544.5614	−544.5576	−544.5566	−544.5886
**As**N(H)N_2C(H)	C_S	0	0	−2438.9331	−2438.8924	−2438.8883	−2438.8874	−2438.9207

3.2 Induced ring currents

To estimate the aromaticity of the five-membered ring systems $PnN(H)N_2C(H)$ (Pn = N, P, As), the magnetically induced ring current density was computed using the gauge-including magnetically induced current (GIMIC) model,[51,52] as implemented in the GIMIC 2.1.4 code,[56,57] which was used in conjunction with the Gaussian09 interface.[73] Nuclear shielding parameters were computed at the PBE0-D3[74]/def2-TZVP level of theory. Graphical representations of the current density were generated using ParaView's[71] streamline plotting feature. Additionally, NICS(0) $NICS(0)_{zz}$, NICS(1) and $NICS(1)_{zz}$ values[76,77] were computed (PBE/def2-TZVP, using the 6-31G(d,p)[78-87] basis at the ghost atoms), which are often used as indicators for aromaticity. The calculated NICS values nicely agree with the results obtained using the GIMIC method.

Table S3. Integrated ring current susceptibilities and NICS values. A positive sign of the induced ring current implies a net diatropic current.

	**Induced ring current susceptibility [nA/T]**	**NICS(0) [ppm]**	**$NICS(1)_{zz}$ [ppm]**
**N**N(H)N_2C(H)	13.8	−14.7	−38.5
**P**N(H)N_2C(H)	14.1	−15.6	−37.9
**As**N(H)N_2C(H)	14.2	−16.1	−37.1

3.3 XYZ Coordinates

3.3.1 **N**N(H)N_2C(H)

```
C        0.63348      -0.90927      -0.00000
H       -0.01016       2.05401       0.00000
H        1.25921      -1.79513      -0.00000
N       -0.00000       1.03696       0.00000
N       -1.12195       0.31436       0.00000
N       -0.72517      -0.94035      -0.00000
N        1.12570       0.33143       0.00000
```

3.3.2 **P**N(H)N_2C(H)

```
C        0.07957       1.19087      -0.00000
H       -0.11365      -2.12908       0.00000
H        0.00888       2.27684      -0.00000
N       -0.00000      -1.11725       0.00000
N        1.27672      -0.68043       0.00000
N        1.31192       0.61829       0.00000
P       -1.23287       0.06418      -0.00000
```

3.3.3 **As**N(H)N_2C(H)

```
C         1.22728      -0.44963       0.00000
H        -2.17215      -0.49823       0.00000
H         2.31278      -0.36186       0.00000
N        -1.15318      -0.50890       0.00000
N        -0.61043      -1.72899       0.00000
N         0.69156      -1.68992       0.00000
As        0.00000       0.94098      -0.00000
```

4 Structure elucidation

X-ray structure determination:

Single crystal X-ray diffraction data was collected on a *Bruker D8 Venture* fitted with a Photon II CMOS Detector with Mo K_α radiation (λ = 0.71073 Å) from an *IμS* micro-source, performing φ-and ω-scans. Data collection and processing was handled using the *Bruker APEX3* and *Bruker APEX4* software packages.[88,89] Absorption corrections were carried out by the multiscan method.[90,91] Structures were solved and refined in Olex2[92] with the SHELX program package.[93,94] All non-hydrogen atoms were refined anisotropically, all hydrogen atoms were included into the model at geometrically calculated positions and refined using a riding model. Structures were checked with checkCIF.[95] Selected crystallographic data can be found in tables S4-S6 below. The representation of molecular structures was done using the program DIAMOND 4.2.2.[96] Some remaining crystallographic problems are commented on in the respective .*cif* file and in captions to the illustrations of the structures in Figure S14-S23. The CCDC entries 2421127 (**1c**), 2421123 (**1d**), 2421121 (**2a**), 2421125 (**2b**), 2421122 (**2c**), 2421124 (**2e**), 2421128 (**2f**), 2421129 (**2g**) and 2421126 (**2i**) contain the supplementary crystallographic data for this article. These data can be obtained free of charge from The Cambridge Crystallographic Data Centre *via* www.ccdc.cam.ac.uk/structures.

Table S4: Crystallographic details of **1c**, **1d**, **2a** and **2b**.

**Compound**	**1c**	**1d**	**2a**	**2b**
Chem. Formula	$C_{10}H_{13}AsCl_2$	$C_{16}H_{25}AsCl_2$	$C_{11}H_{16}AsN_3Si$	$C_{14}H_{12}AsN_3$
Formula weight [g/mol]	279.02	363.18	293.28	297.19
Colour	colourless	colourless	colourless	colourless
Crystal system	monoclinic	triclinic	monoclinic	monoclinic
Space group	$P2_1/n$	$P\bar{1}$	$P2_1/c$	$P2_1/c$
a [Å]	8.64750(10)	9.5234(7)	11.1008(2)	12.1065(9)
b [Å]	8.53770(10)	9.9594(8)	5.78310(10)	4.5269(4)
c [Å]	15.4366(3)	20.4691(15)	21.2959(5)	22.8439(17)
α [°]	90	96.850(3)	90	90
β [°]	90.5589(6)	100.313(3)	99.4390(10)	92.336(3)
γ [°]	90	110.562(2)	90	90
V [Å^3]	1134.32(3)	1753.1(2)	1348.62(5)	1250.92(17)
Z	4	4	4	4
$\rho_{calcd.}$ [g/cm^3]	1.634	1.376	1.444	1.578
μ [mm^{-1}]	3.420	2.231	2.588	2.701
T [K]	100	100	100	100
Measured reflections	9608	12130	12859	30330
Independent reflections	2320	12130	2758	3731
Reflections with $I > 2\sigma(I)$	2136	11250	2377	2882
R_{int}	0.0273	- (merged)	0.0481	0.0488
F(000)	560	752	600	600
$R_1(R[F^2>2\sigma(F^2)])$	0.0201	0.0294	0.0267	0.0559
w$R_2(F^2)$	0.0491	0.0882	0.0560	0.1028
GooF	1.085	1.138	1.081	1.092
No. of Parameters	121	349	148	185
CCDC #	2421127	2421123	2421121	2421125

Table S5: Crystallographic details of **2c**, **2e**, **2f** nd **2g**.

**Compound**	**2c**	**2e**	**2f**	**2g**
Chem. Formula	$C_{17}H_{18}AsN_3$	$C_{13}H_{20}AsN_3Si$	$C_{19}H_{22}AsN_3$	$C_{19}H_{22}AsN_3$
Formula weight [g/mol]	339.26	321.33	367.31	367.31
Colour	colourless	colourless	colourless	colourless
Crystal system	monoclinic	monoclinic	monoclinic	monoclinic
Space group	$P2_1/n$	$P2_1/n$	$P2_1/c$	$C2/c$
a [Å]	11.0015(2)	8.7864(7)	10.9961(2)	29.752(6)
b [Å]	8.04380(10)	11.5039(10)	8.4381(2)	16.915(3)
c [Å]	35.6994(7)	15.5526(13)	19.0197(4)	11.451(2)
α [°]	90	90	90	90
β [°]	98.6423(7)	97.895(3)	91.2443(8)	108.10(3)
γ [°]	90	90	90	90
V [Å^3]	3123.31(9)	1557.1(2)	1764.35(6)	5478(2)
Z	8	4	4	12
$\rho_{calcd.}$ [g/cm^3]	1.443	1.371	1.383	1.336
μ [mm^{-1}]	2.173	2.248	1.930	1.865
T [K]	100	100	100	100
Measured reflections	64123	62757	10514	87422
Independent reflections	7187	3328	3089	5031
Reflections with $I > 2\sigma(I)$	6113	2970	2581	4221
R_{int}	0.0694	0.0406	0.0288	0.1034
F(000)	1392	664	760	2280
$R_1(R[F^2>2\sigma(F^2)])$	0.0457	0.0280	0.0389	0.0288
$wR_2(F^2)$	0.1011	0.0629	0.1049	0.0645
GooF	1.130	1.114	1.050	1.034
No. of Parameters	385	169	214	334
CCDC #	2421122	2421124	2421128	2421129

Table S6: Crystallographic details of **2i**.

**Compound**	**2i**
Chem. Formula	$2(C_{34}H_{36}AsN_3)*C_5H_{12}$
Formula weight [g/mol]	1195.30
Colour	colourless
Crystal system	monoclinic
Space group	*C2/c*
a [Å]	44.558(3)
b [Å]	8.5288(5)
c [Å]	16.1255(10)
α [°]	90
β [°]	92.575(2)
γ [°]	90
V [Å^3]	6122.0(6)
Z	4
$\rho_{calcd.}$ [g/cm^3]	1.297
μ [mm^{-1}]	1.140
T [K]	100
Measured reflections	46212
Independent reflections	5825
Reflections with $I > 2\sigma(I)$	4436
R_{int}	0.0478
F(000)	2520
$R_1(R[F^2>2\sigma(F^2)])$	0.0736
$wR_2(F^2)$	0.1804
GooF	1.072
No. of Parameters	377
CCDC #	2421126

Figure S14: Ellipsoid representation of **1c**. The thermal ellipsoids are set at a 50% probability level.

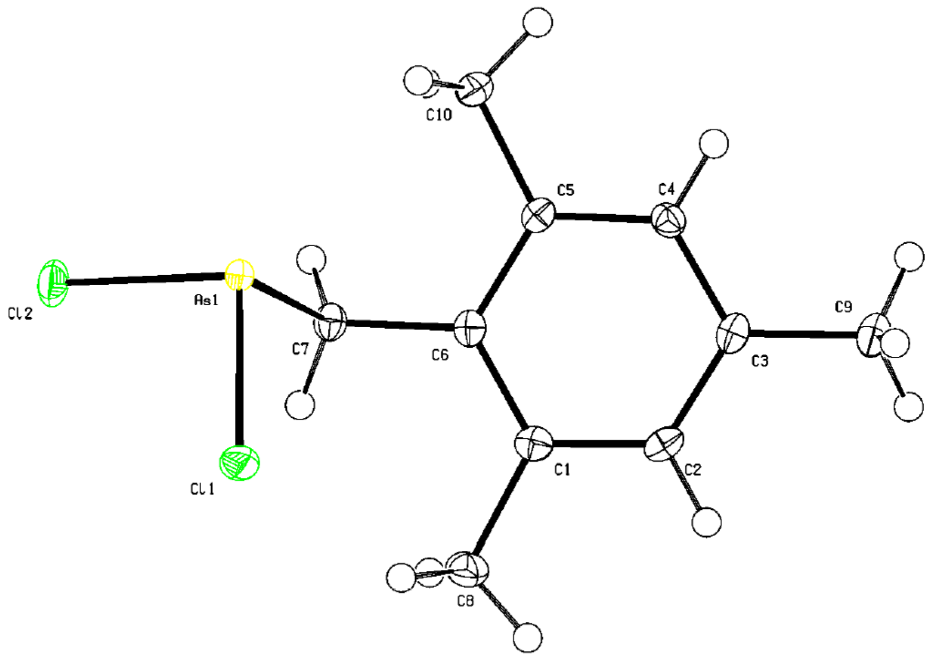

Figure S15: Ellipsoid representation of **1d**. The structure was refined as a twin with two domains with the second domain contributing with a BASF of 0.27305.The thermal ellipsoids are set at a 50% probability level. The crystal contains two molecules per asymmetric unit and one of them exhibits a 2-fold rotational disorder of the 2,4,6-tri*iso*propylphenyl substituent. Thermal constraints (EADP) were employed to the disordered atoms which are close together/partially overlapping.

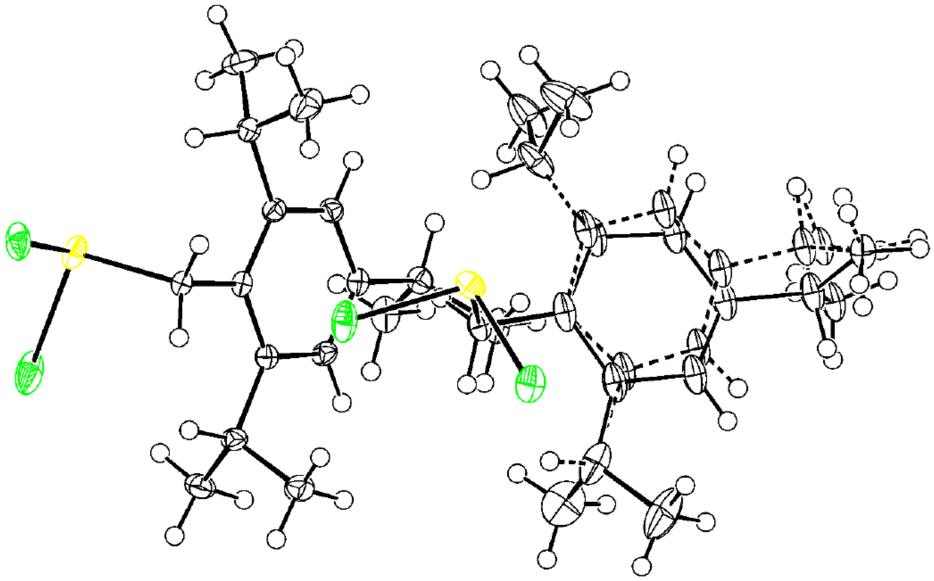

Figure S16: Ellipsoid representation of **2a**. The thermal ellipsoids are set at a 50% probability level.

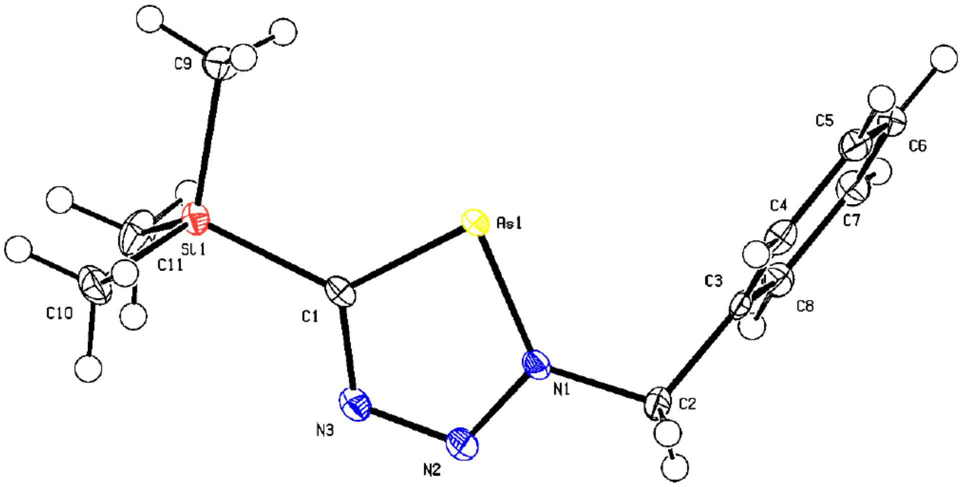

Figure S17: Ellipsoid representation of **2b**. The thermal ellipsoids are set at a 50% probability level. The crystal contains one molecule per asymmetric unit which exhibits a 2-fold rotational disorder of the central N_3CAs-ring. Thermal constraints (EADP) were employed to the disordered atoms N3/N1B.

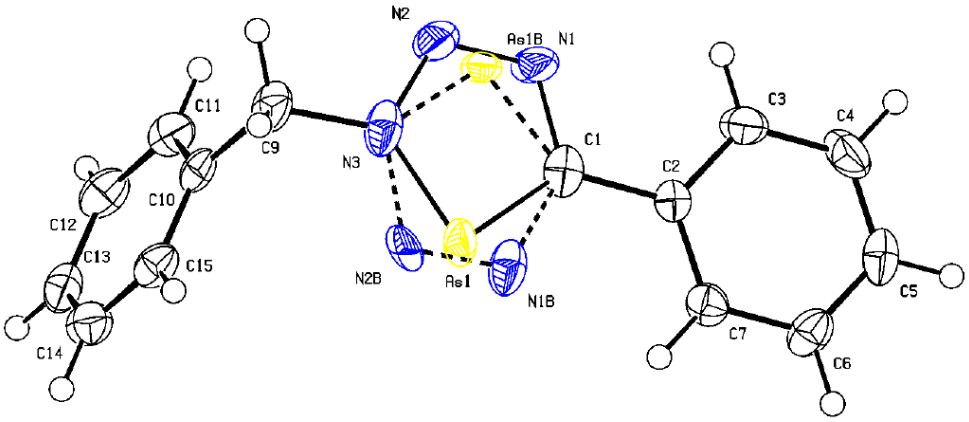

Figure S18: Ellipsoid representation of **2c**. The thermal ellipsoids are set at a 50% probability level.

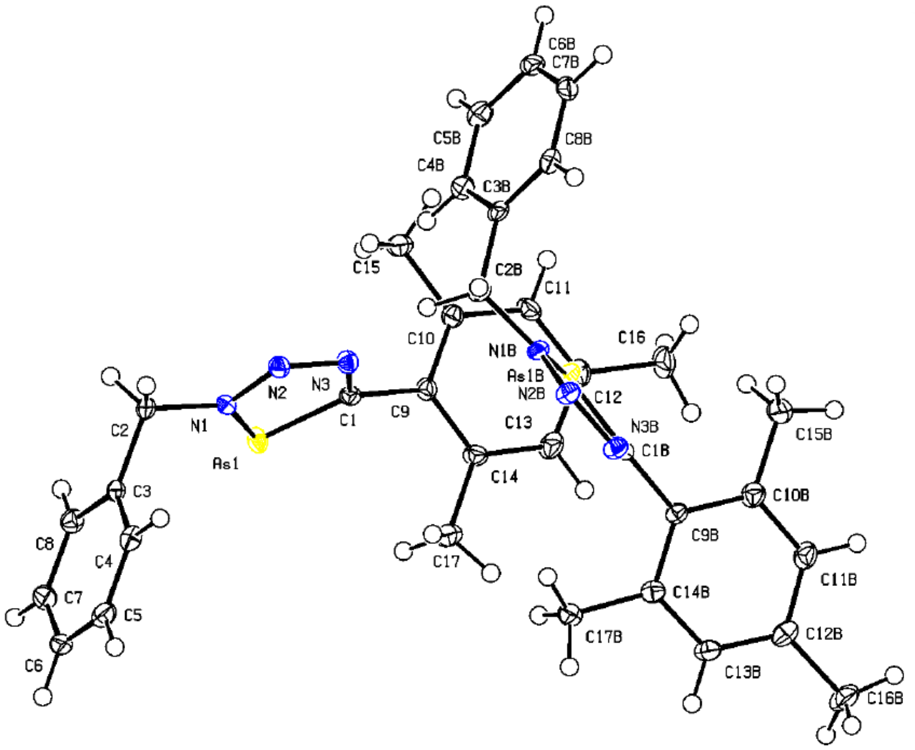

Figure S19: Ellipsoid representation of **2e**. The thermal ellipsoids are set at a 50% probability level.

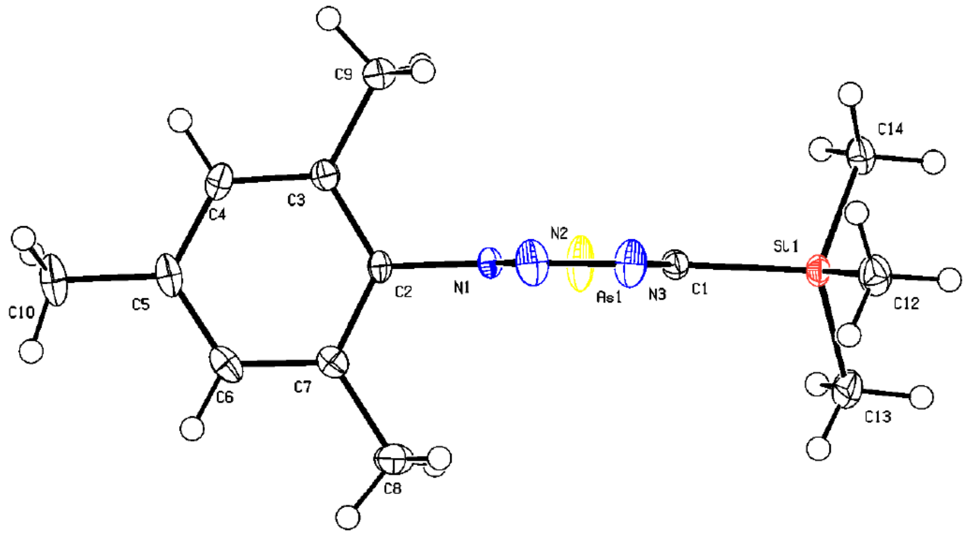

Figure S20: Ellipsoid representation of **2f**. The thermal ellipsoids are set at a 50% probability level.

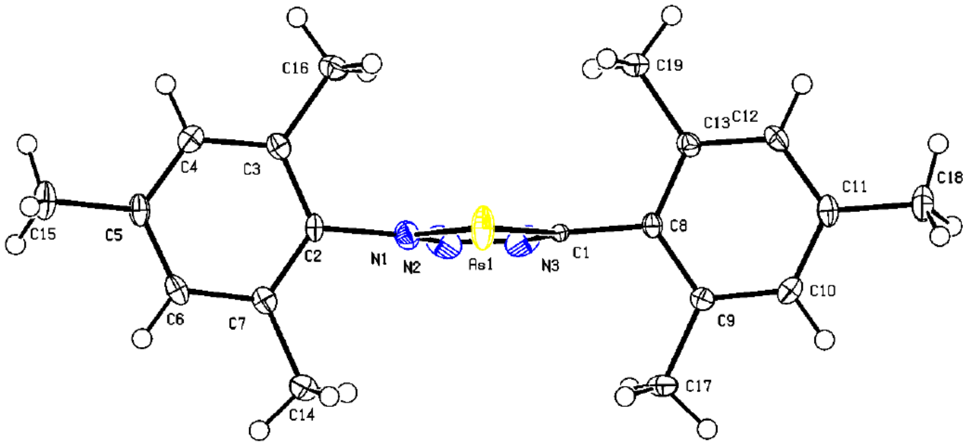

Figure S21: Ellipsoid representation of **2g**. The thermal ellipsoids are set at a 50% probability level. The crystal contains one and a half molecule per asymmetric and one of the exhibits a 2-fold rotational disorder of the central N_3CAs-ring. This disorder was resolved by a Part −1 instruction. No further restrains were applied

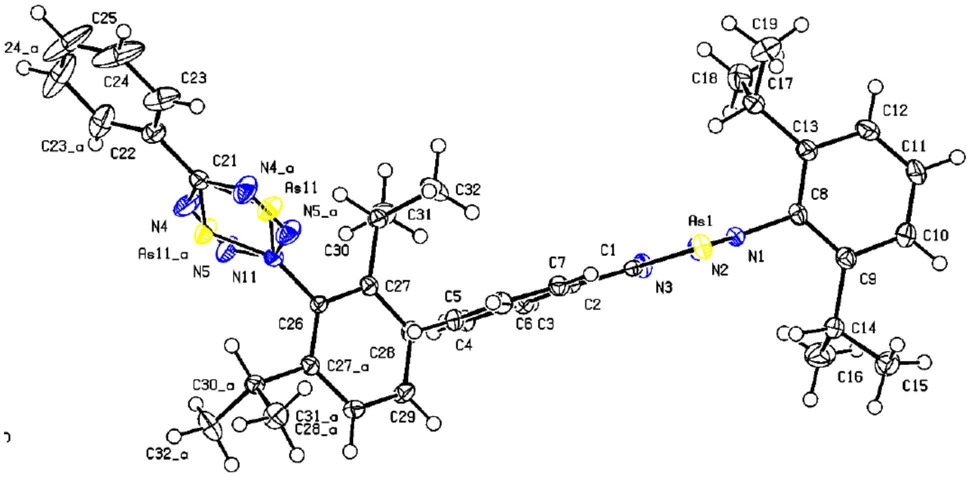

Figure S22: Ellipsoid representation of **2i**. The thermal ellipsoids are set at a 50% probability level.

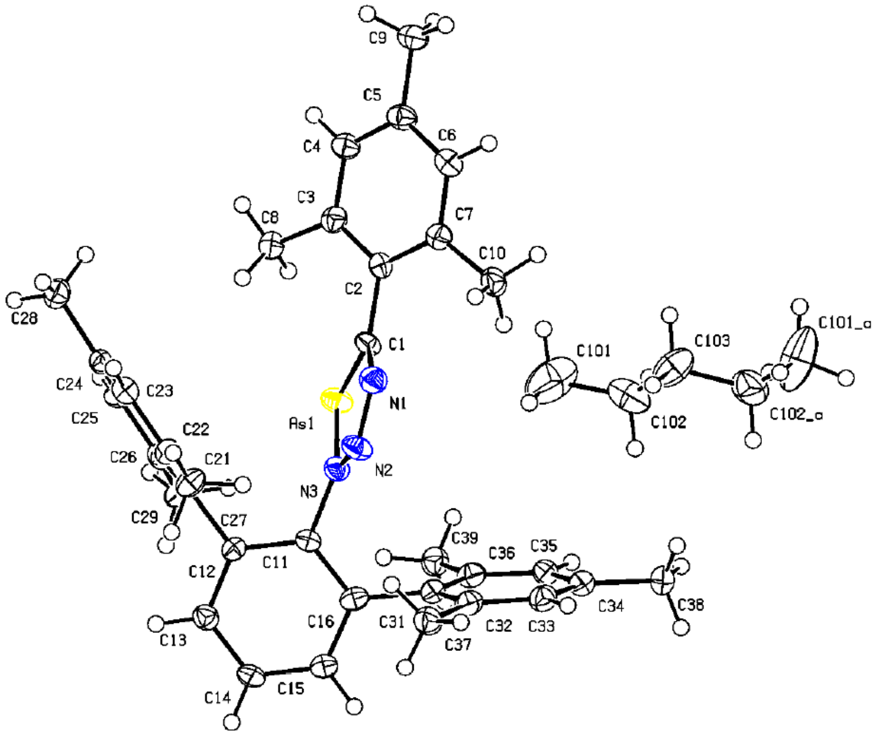

5 References

(as cited in manuscript)

[58] E. W. Abel, D. A. Armitage, G. R. Willey, *J. Chem. Soc.* **1965**, 57.

[59] H. Andringa, Y. A. Heus-Kloos, L. Brandsma, *J. Organomet. Chem.* **1987**, *336*, C41–C43.

[60] K. Moriya, M. Simon, R. Mose, K. Karaghiosoff, P. Knochel, *Angew. Chemie Int. Ed.* **2015**, *54*, 10963–10967.

[61] *Gaussian 16, Revision C.02*, M. J. Frisch, G. W. Trucks, H. B. Schlegel, G. E. Scuseria, M. A. Robb, J. R. Cheeseman, G. Scalmani, V. Barone, G. A. Petersson, H. Nakatsuji, X. Li, M. Caricato, A. V. Marenich, J. Bloino, B. G. Janesko, R. Gomperts, B. Mennucci, H. P. Hratchian, J. V. Ortiz, A. F. Izmaylov, J. L. Sonnenberg, D. Williams-Young, F. Ding, F. Lipparini, F. Egidi, J. Goings, B. Peng, A. Petrone, T. Henderson, D. Ranasinghe, V. G. Zakrzewski, J. Gao, N. Rega, G. Zheng, W. Liang, M. Hada, M. Ehara, K. Toyota, R. Fukuda, J. Hasegawa, M. Ishida, T. Nakajima, Y. Honda, O. Kitao, H. Nakai, T. Vreven, K. Throssell, J. A. Montgomery, Jr., J. E. Peralta, F. Ogliaro, M. J. Bearpark, J. J. Heyd, E. N. Brothers, K. N. Kudin, V. N. Staroverov, T. A. Keith, R. Kobayashi, J. Normand, K. Raghavachari, A. P. Rendell, J. C. Burant, S. S. Iyengar, J. Tomasi, M. Cossi, J. M. Millam, M. Klene, C. Adamo, R. Cammi, J. W. Ochterski, R. L. Martin, K. Morokuma, O. Farkas, J. B. Foresman, and D. J. Fox, Gaussian, Inc., Wallingford CT, **2019**.

[62] J. P. Perdew, K. Burke, M. Ernzerhof, *Phys. Rev. Lett.* **1996**, *77*, 3865–3868.

[63] J. P. Perdew, K. Burke, M. Ernzerhof, *Phys. Rev. Lett.* **1997**, *78*, 1396–1396.

[64] S. Grimme, J. Antony, S. Ehrlich, H. Krieg, *J. Chem. Phys.* **2010**, *132*, 154104.

[65] S. Grimme, S. Ehrlich, L. Goerigk, *J. Comput. Chem.* **2011**, *32*, 1456–1465.

[66] F. Weigend, R. Ahlrichs, *Phys. Chem. Chem. Phys.* **2005**, *7*, 3297.

[67] F. Weigend, *Phys. Chem. Chem. Phys.* **2006**, *8*, 1057–1065.

[68] F. London, *J. Phys. Radium* **1937**, *8*, 397–409.

[69] R. McWeeny, *Phys. Rev*. **1962**, *126*, 1028–1034.

[70] R. Ditchfield, *Mol. Phys.* **1974**, *27*, 789–807.

[71] K. Wolinski, J. F. Hinton, P. Pulay, *J. Am. Chem. Soc.* **1990**, *112*, 8251–8260.

[72] J. R. Cheeseman, G. W. Trucks, T. A. Keith, M. J. Frisch, *J. Chem. Phys.* **1996**, *104*, 5497–5509.

[73] M. Rauhalahti, S. Taubert, D. Sundholm, V. Liégeois, *Phys. Chem. Chem. Phys.* **2017**, *19*, 7124–7131.

[74] C. Adamo, V. Barone, *J. Chem. Phys.* **1999**, *110*, 6158–6170.

[75] J. Ahrens, B. Geveci, C. Law, in *Vis. Handb., Elsevier*, **2005**, pp. 717–731.

[76] P. von R. Schleyer, C. Maerker, A. Dransfeld, H. Jiao, N. J. R. van Eikema Hommes, *J. Am. Chem. Soc.* **1996**, *118*, 6317–6318.

[77] H. Fallah-Bagher-Shaidaei, C. S. Wannere, C. Corminboeuf, R. Puchta, P. v. R. Schleyer, *Org. Lett.* **2006**, *8*, 863–866.

[78] R. Ditchfield, W. J. Hehre, J. A. Pople, *J. Chem. Phys.* **1971**, *54*, 724–728.

[79] W. J. Hehre, R. Ditchfield, J. A. Pople, *J. Chem. Phys.* **1972**, *56*, 2257–2261.

[80] P. C. Hariharan, J. A. Pople, *Theor. Chim. Acta* **1973**, *28*, 213–222.

[81] P. C. Hariharan, J. A. Pople, *Mol. Phys.* **1974**, *27*, 209–214.

[82] M. S. Gordon, *Chem. Phys. Lett.* **1980**, *76*, 163–168.

[83] M. M. Francl, W. J. Pietro, W. J. Hehre, J. S. Binkley, M. S. Gordon, D. J. DeFrees, J. A. Pople, *J. Chem. Phys.* **1982**, *77*, 3654–3665.

[84] R. C. Binning Jr., L. A. Curtiss, *J. Comput. Chem.* **1990**, *11*, 1206–1216.

[85] J.-P. Blaudeau, M. P. McGrath, L. A. Curtiss, L. Radom, *J. Chem. Phys.* **1997**, *107*, 5016–5021.

[86] V. A. Rassolov, J. A. Pople, M. A. Ratner, T. L. Windus, *J. Chem. Phys.* **1998**, *109*, 1223–1229.

[87] V. A. Rassolov, M. A. Ratner, J. A. Pople, P. C. Redfern, L. A. Curtiss, *J. Comput. Chem.* **2001**, *22*, 976–984.

[88] Bruker (**2019**) *APEX III*. Bruker AXS Inc., Madison, Wisconsin, USA.

[89] Bruker (**2021**). *APEX4, SAINT and SADABS.* Bruker AXS Inc., Madison, Wisconsin, USA.

[90] G. M. Sheldrick, SADABS; University of Göttingen: Germany, **1996**.

[91] P. Coppens, *The Evaluation of Absorption and Extinction in Single-Crystal Structure Analysis. Crystallographic Computing,* Copenhagen, Muksgaard **1979**.

[92] O. V. Dolomanov, L. J. Bourhis, R. J. Gildea, J. a. K. Howard, H. Puschmann, *J. Appl. Cryst.* **2009**, *42*, 339–341.

[93] G. M. Sheldrick, *Acta Crystallogr., Sect. A: Found. Crystallogr.* **2008**, A64, 112.

[94] G. M. Sheldrick, *Acta Crystallogr., Sect. C: Struct. Chem*. **2015**, C71, 3.

[95] The facility “CheckCIF,” can be found at http://checkcif.iucr.org.

[96] *Diamond - Crystal and Molecular Structure Visualization; Crystal Impact* - Dr. H. Putz & Dr. K. Brandenburg GbR: Bonn, Germany, **2019**.
